# SNAPpa: A Photoactivatable SNAP-tag for the Spatiotemporal Control of Protein Labeling

**DOI:** 10.1021/jacsau.5c00603

**Published:** 2025-07-17

**Authors:** Sabrina Mandl, Barbara Maiwald, Elena Adlmanninger, Ramona Birke, Sandra Schlee, Adam Pruška, Philipp Bittner, Renato Zenobi, Tolga Soykan, Gerti Beliu, Johannes Broichhagen, Andrea Hupfeld

**Affiliations:** 1 Institute of Biophysics and Physical Biochemistry, Regensburg Center for Biochemistry, 98913 University of Regensburg, Universitätsstraße 31, Regensburg D-93053, Germany; 2 28417 Leibniz-Forschungsinstitut für Molekulare Pharmakologie (FMP), Robert-Roessle-Str. 10, Berlin 13125, Germany; 3 Department of Chemistry and Applied Biosciences, 27219 ETH Zurich, Zurich CH-8093, Switzerland; 4 Regensburg Center for Ultrafast Nanoscopy (RUN), Bioimaging, Faculty of Chemistry and Pharmacy, 98913 University of Regensburg, Regensburg 93040, Germany; 5 Rudolf Virchow Center, Research Center for Integrative and Translational Bioimaging, 9190 University of Würzburg, Würzburg 97080, Germany

**Keywords:** fluorescent label, photocage, photocontrol, protein engineering, self-labeling protein tag, unnatural amino acids

## Abstract

SNAP-tag is one of the most commonly used self-labeling protein tags for cell imaging studies. To achieve selective spatiotemporal imaging of cells, we set out to engineer a photoactivatable SNAP-tag. For this, we incorporated the well-established and readily available photocaged unnatural amino acid *o*-nitrobenzyl-*O*-tyrosine (ONBY) into all three tyrosine positions of SNAP. In-gel imaging analysis and fluorescence polarization measurements revealed that placing ONBY in position Y114 of the SNAP-tag facilitates the most effective and most efficient photoactivation of the irreversible self-labeling reaction with (sulfonated) benzyl guanine substrates, which is why we dubbed this photoactivatable SNPA-tag variant “SNAPpa”. To demonstrated its potential for live-cell imaging, we further tested SNAPpa in HEK293 cells, either fused to a nuclear localization domain for intracellular imaging or fused to either a transmembrane region or the glucagon-like peptide 1 receptor for extracellular imaging. Each SNAPpa construct produced no fluorescence signal when ONBY remained in its photocaged state by keeping the cells in the dark. However, a clear fluorescence signal appeared after light-induced decaging of ONBY. Applying a localized light beam thereby highlighted the precise spatiotemporal control of cell imaging. In conclusion, SNAPpa can be used for the efficient light-induced activation of fluorescence labeling and can be easily established, readily implemented and effectively combined with the broad repertoire of substrates that is already available for SNAP.

## Graphical abstract

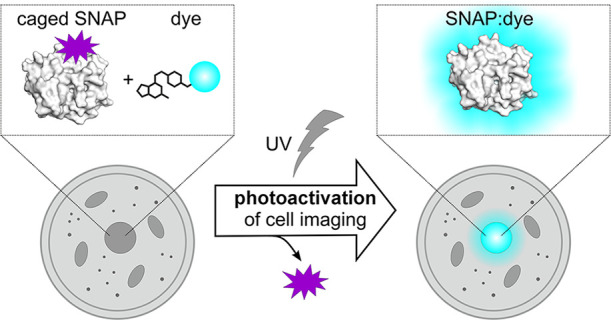

## Introduction

Fluorescence labeling is a simple and widespread method for functional investigation and localization or tracking of proteins. It has been realized through the discovery of fluorescent proteins such as green fluorescent protein (GFP) that are fused to a protein of interest (POI). Major drawbacks of these fluorescent proteins are the required maturation time of the fluorophore and its photolability, which initiated the development of self-labeling protein tags.

One of the most prominently used self-labeling protein tags is the SNAP-tag.^1–4^ This protein is in itself nonfluorescent and originates from the human *O* ^6^-alkylguanine-DNA alkyltransferase.^1^ It reacts with *O* ^6^-alkyl- or *O* ^6^-benzylguanine (BG) in a one-step suicide reaction covalently functionalizing a cysteine residue in the active site with either the alkyl or benzyl group, respectively, and setting free the guanine moiety.^5,6^ By attaching a fluorophore to the benzyl moiety, this reaction allows for the irreversible fluorescence self-labeling of SNAP and any protein to which it is fused (Figure 1A). Due to various steps of engineering, the final SNAP-tag has lost its affinity toward DNA, is produced with high expression levels in pro- and eukaryotic cells, is stable against proteases, and is thermally stable at ambient and physiological temperatures.^7^ These attributes make it primarily valuable for cell studies, although it is also used in an immobilization strategy for biosensors, biomedical implants, and biocatalysts.^8–12^ Owing to its significance for cell biology, a broad repertoire of fluorescent BG substrates has been developed with different performances on membrane (im)­permeability, fluorogenicity, or sensing abilities.^13^

**Figure 1:**
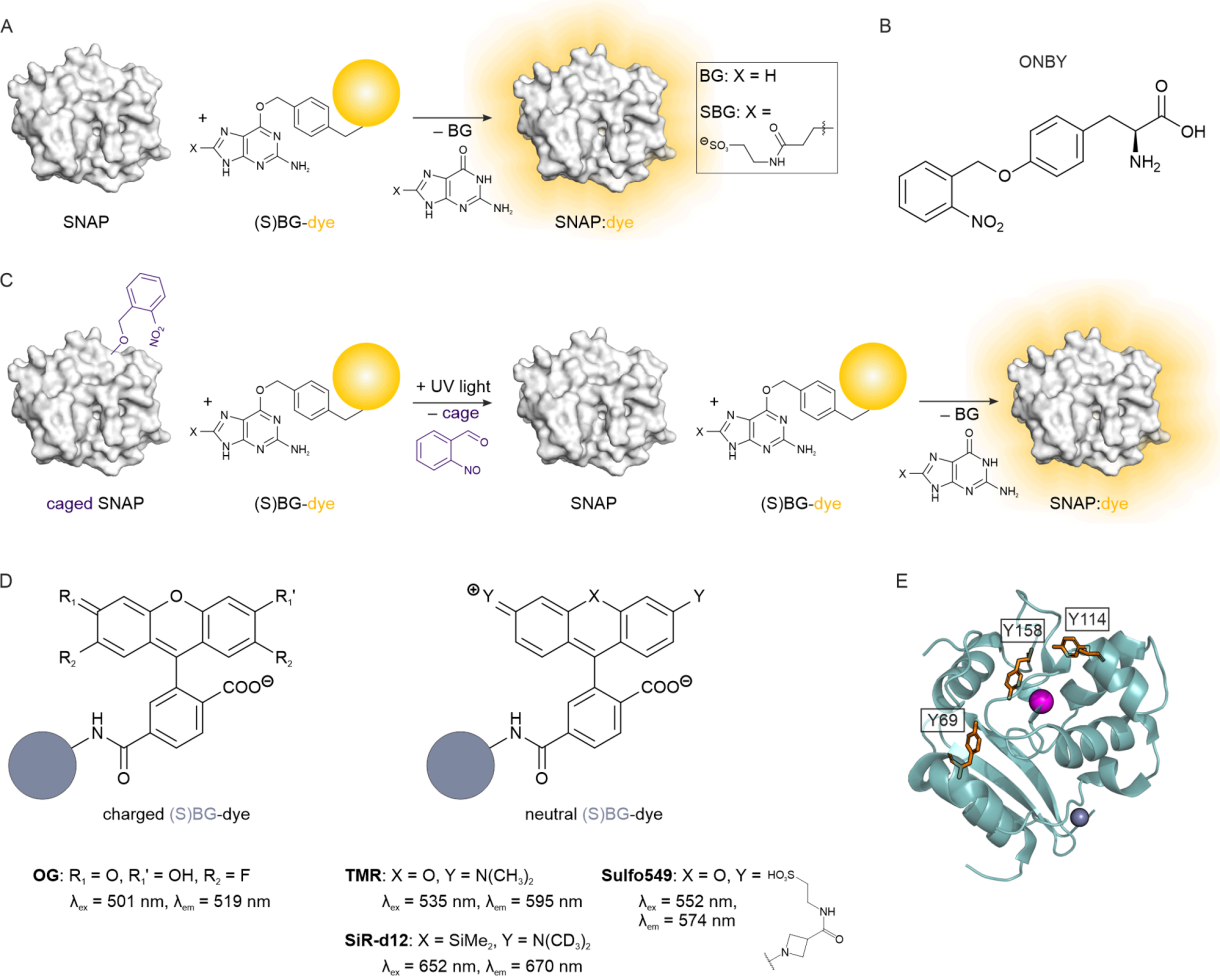
Design of the photocontrol in SNAP-tag. (A) Self-labeling reaction of the SNAP-tag with (S)­BG substrates attached to fluorescent dyes. (B) Unnatural amino acid *o*-nitrobenzyl-*O*-tyrosine (ONBY). (C) Photoactivation of the SNAP self-labeling reaction via incorporation of ONBY and light-induced release of the molecular cage. (D) Representative charged and neutral xanthene-based fluorescent SNAP labels used in this study. Note that sulfonation either on BG or xanthene makes the SNAP label cell-impermeable. (E) All tyrosine positions in SNAP (orange sticks) for the incorporation of ONBY are in proximity to the reactive cysteine in the active site (pink sphere). Grayish blue sphere: Zn^2+^; PDB ID 3kzz.

Another desired advancement for self-labeling protein tags, including the SNAP-tag, is the spatiotemporal control of the labeling reaction. This is particularly interesting for cell studies since it would make it possible to track the POI at particular time points and/or its movement within the cell or to separate protein pools for instance by pulse-chasing. To this end, UV photoactivatable BG substrates have been designed in the past decade.^14–16^ In a similar vein, substrates delivering a caged fluorophore have been reported for super-resolution imaging^17,18^ and single-particle tracking.^19^ However, a more versatile and therefore highly innovative approach would be the targeted photocontrol of the SNAP-tag itself allowing us to still make use of the vast repertoire of fluorescent probes and eradicating the diffusion of unleashed substrates to different sites and covalent saturation of all tags. This can be realized by the site-specific incorporation of photosensitive unnatural amino acids (UAAs) in response to a reprogrammed amber codon via an orthogonal, engineered aminoacyl-tRNA synthetase/tRNA (aaRS/tRNA) pair, an approach known as photoxenoprotein engineering.^20–22^ The most efficient photosensitive UAAs for the photoactivation of the suicide self-labeling reaction of the SNAP-tag are certainly those that contain a photolabile protecting group that is irreversibly cleaved off by irradiation with light of a specific wavelength to release a reactive natural amino acid. One of the most frequently utilized photocaged UAAs is *o*-nitrobenzyl-*O*-tyrosine (ONBY; Figure 1B), in which the protecting group *o*-nitrobenzaldehyde is cleaved off upon irradiation with UV to blue light and the natural l-tyrosine remains as a product.^23,24^

In this study, we set out to design and apply a photoactivatable SNAP-tag, dubbed SNAPpa, for the highly desired spatiotemporal control of fluorescent labeling with various charged and neutral xanthene-based fluorescent dyes in cell studies (Figure 1C,D). To this end, we incorporated the well-established photocaged UAA ONBY at three tyrosine positions and evaluated the ability of the resulting variants to efficiently control the suicide reaction of the SNAP-tag. We then demonstrated the applicability of the best SNAP variant in various live-cell imaging studies.

## Results

### Design, Production, and Characterization of SNAP-ONBY Variants

For the engineering of a photoactivatable SNAP-tag, we decided to incorporate ONBY only into tyrosine positions to circumvent point mutations that could alter the SNAP reactivity. Crystal structure analysis of SNAP showed that the three tyrosines contained in SNAP are positioned in proximity to the BG binding site (Figure 1E). While Y69 is ∼10 Å distant from the reactive cysteine, Y114 and Y158 are closer with ∼8 and ∼6 Å, respectively. Thus, we incorporated ONBY into these three positions of the SNAP-tag. Since the SNAP-tag is often used in combination with another self-labeling protein such as HaloTag (HT7) for cell imaging studies, we also inserted ONBY into a SNAP-HaloTag (HT7) fusion protein (Figure 2A). For the sake of brevity, we use the terms SNAP and SNAP-HT7 for the constructs without ONBY and refer to SNAP­(-HT7) constructs that contain ONBY at position Y69, Y114, or Y158 with the terms SNAP-Y69ONBY­(-HT7), SNAP-Y114ONBY­(-HT7), or SNAP-Y158ONBY­(-HT7), respectively. We produced each construct via heterologous gene expression in *Escherichia coli* (*E. coli*) and subsequent purification by metal affinity as well as size-exclusion chromatography. For this purpose, we employed a previously optimized aaRS/tRNA pair for the efficient and selective incorporation of ONBY that is orthogonal in prokaryotic expression systems.^25^ While repeated trials to obtain SNAP-Y69ONBY­(-HT7) variants were unsuccessful, all other ONBY variants achieved similar or even up to six-fold higher yields per liter expression medium compared to SNAP and SNAP-HT7 likely due to the nutrition-rich terrific broth (TB) medium used for expression with ONBY. Moreover, the purity was >90% for SNAP variants and >80% for SNAP-HT7 variants, as confirmed by SDS-PAGE (Figure S1A). We then set out to verify the incorporation of ONBY and the structural integrity of all variants using native mass spectrometry (MS; Figures S2 and S3). The data indicated that ONBY was present in all variants, except SNAP and SNAP-HT7. Notably, we also detected the reduced form of ONBY named *o*-aminobenzyl-*O*-tyrosine (OABY), which is common for the production in prokaryotic expression hosts.^26^ While the native mass spectra demonstrated that all SNAP variants were Zn^2+^ binding monomers (Figure S2) confirming previous results,^27^ they showed that all SNAP-HT7 variants lacked Zn^2+^ and that SNAP-Y114ONBY-HT7 and SNAP-Y158ONBY-HT7 oligomerized (Figure S3). Although we have no indication how this effect was caused, it might be an artifact of the high protein concentrations used in vitro and might hence be irrelevant for cell studies, in which the protein concentration is generally low. We further analyzed the structural integrity in circular dichroism measurements confirming that the secondary structure was well-preserved in comparison to SNAP and SNAP-HT7 (Figure S4A,B). Finally, the ONBY variants showed a similar thermal stability as SNAP and SNAP-HT7 with denaturation midpoints of ∼64 and ∼61 °C, respectively, as determined using differential scanning calorimetry (Figure S4C,D).

**Figure 2:**
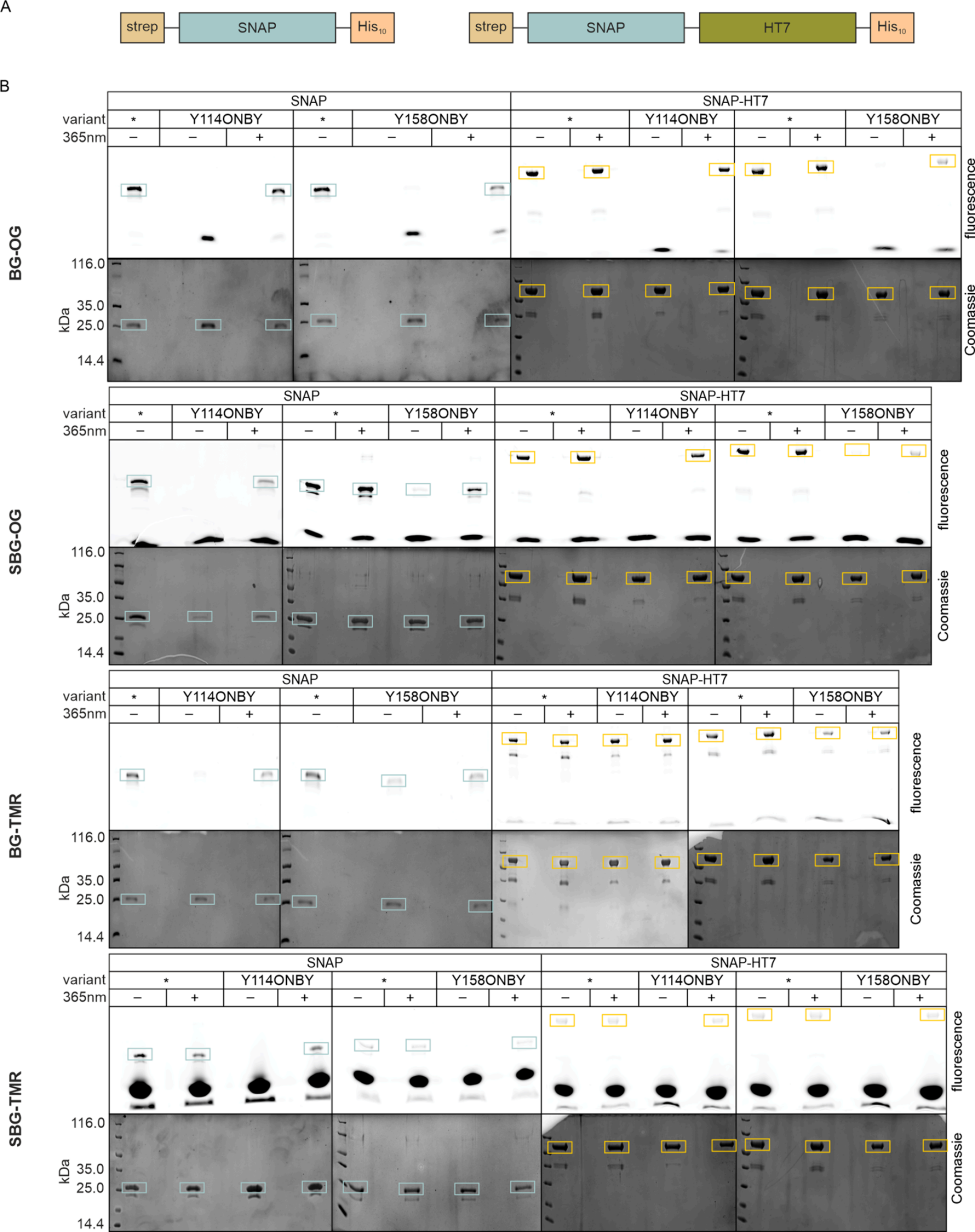
Self-labeling activity of SNAP­(-HT7) ONBY variants using in-gel fluorescence analysis. (A) SNAP and SNAP-HT7 constructs used for in vitro experiments contain an n-terminal strep-tag and a c-terminal His-tag for the protein purification after heterologous gene expression in *Escherichia coli*. (B) SDS-PAGE analysis of SNAP­(-HT7) variants in the presence of (S)­BG substrates indicate that nonirradiated (−), photocaged proteins forfeit the ability of self-labeling and regain it after irradiation with 365 nm for 2 min (+). *: no ONBY incorporated; blue boxes: SNAP proteins have molecular weights of ∼24 kDa; orange boxes: SNAP-HT7 proteins with molecular weights of ∼59 kDa. The lowest bands in the fluorescence panels indicate an unretained, free substrate. Additional bands at ∼23 kDa for all SNAP variants and ∼36 kDa for all SNAP-HT7 variants resulted from proteolytic cleavage as previously observed.^31^ Note: vertical lines separate the individual gels, which all start with one lane of a low-molecular-weight marker.

### The Presence of ONBY in the Active Site Prevents the Self-Labeling Reaction

Next, we set out to test the SNAP reaction of all ONBY variants in their nonirradiated, photocaged state and in their irradiated, decaged state. For this purpose, we selected representative charged and neutral fluorescent dyes, namely, Oregon Green (OG) and tetramethylrhodamine (TMR; Figure 1D), which are available in sufficient quantities for extended biophysical studies.^13,28,29^ To extend our repertoire with cell-impermeable SNAP-tag labels for the imaging of cell surface proteins, we chose to include the respective SBG substrates,^13^ which are sulfonated at the BG-C8 position (Figure 1A,C). This modification is expected to influence the self-labeling reaction and hence possibly the photoactivation more than sulfonation of the dye, which resides outside the active site of SNAP.^30^ We started our investigation with an in-gel fluorescence analysis, in which the self-labeling of SNAP­(-HT7) variants was evaluated by SDS-PAGE. Fluorescence imaging of the gel allowed the detection of unretained, free substrates and substrates retained by the covalent bond to SNAP­(-HT7) (upper panels in Figure 2B). As a control, the gel was subsequently stained with Coomassie to visualize the protein irrespective of its labeling status (bottom panels). Initial tests were performed with different concentrations of SNAP, which verified that the measured fluorescence intensities of the retained signals in the SDS-PAGE reliably reflected the amounts of self-labeled protein (Figure S5A). Hence, in-gel fluorescence analysis provides a simple method to screen for differences in the self-labeling efficiencies of the nonirradiated, photocaged (“–”) and irradiated, decaged (“+”) ONBY variants. Importantly, irradiation of SNAP and SNAP-HT7 prior to SDS-PAGE did not affect the reaction with its substrates. Remarkably, photocaged SNAP-Y114ONBY­(-HT7) forfeited the ability to bind BG-OG, SBG-OG, BG-TMR, and SBG-TMR as indicated by the absence of fluorescence or minor fluorescence signals (Figure 2B and Figure S5B). The only exception was the reaction between photocaged SNAP-Y114ONBY-HT7 and BG-TMR, which produced a substantial fluorescence intensity in the SDS-PAGE. Irradiation-induced decaging resulted in high intensities for all of the SNAP-Y114ONBY­(-HT7) and BG substrate combinations. In comparison to SNAP-Y114ONBY­(-HT7), photocaged SNAP-Y158ONBY­(-HT7) showed stronger fluorescence signals that were still weak for BG-OG, SBG-OG, and SBG-TMR but quite pronounced for BG-TMR (Figure 2B and Figure S5B). Moreover, irradiation-induced decaging led to similar or slightly lower signal intensities than that for SNAP-Y114ONBY­(-HT7). These results implicated that the self-labeling of SNAP can generally be hampered by the incorporation of ONBY close to the reactive cysteine and restored after irradiation-induced decaging.

### Photoactivation of SNAP Is Highly Efficient with ONBY in Position Y114

In-gel fluorescence analysis provided the first insight into the photoactivation of SNAP self-labeling with ONBY in positions Y114 and Y158. However, to further evaluate which SNAP-ONBY variant is most efficient, we set out to compare the self-labeling rates before and after irradiation, which provides more detailed information on the binding of the BG substrates.

Previous studies have shown that the mobility of the BG substrates is restricted upon binding to SNAP,^13,30^ which influences their fluorescent properties. The resulting difference can be resolved via the measurement of the fluorescence polarization. An increase and saturation of the corresponding signal over time thereby indicate the binding of substrates and facilitates the determination of the self-labeling rate *k*_app_ as well as the half-time of binding *t*_1/2_. Initial experiments were performed with SNAP­(-HT7) in combination with all of the BG substrates. Despite repeated measurements under various conditions, we were unable to obtain a binding signal for both TMR substrates. Hence, we determined the *k*_app_ and *t*_1/2_ values for all SNAP­(-HT7) variants only for BG-OG and SBG-OG (Table 1 and Figure S6). For BG-OG, the reaction with SNAP was ∼1.6-fold faster than for SNAP-HT7 with *k*_app_ values of 6.8 × 10^4^ and 4.3 × 10^4^ M^–1^s^–1^, respectively. The reaction with SBG-OG was in overall 4–5 times slower but showed the same behavior of SNAP and SNAP-HT7 with *k*_app_ values of 1.5 × 10^4^ and 0.9 × 10^4^ M^–1^s^–1^, respectively. Moreover, we tested whether irradiation might harm the proteins and consequently affect the binding of the BG substrates. As a result, we measured *k*_app_ values for the binding of (S)­BG-OG on SNAP­(-HT7) that were minimally reduced with factors of 1.0–1.6.

**Table 1:** Self-Labeling Kinetics of SNAP­(-HT7) Variants in Their Nonirradiated, Photocaged (“–”) and Irradiated, Decaged (“+”) State (365 nm, 2 min)

**fluorescence polarization**						
	SNAP	SNAP-Y114ONBY	SNAP-Y158ONBY	SNAP-HT7	SNAP-Y114ONBY-HT7	SNAP-Y158ONBY-HT7
BG-OG (−)						
*k*_app_ [M^–1^s^–1^]	6.8 × 10^4^	n.d.^a^	n.d.^a^	4.3 × 10^4^	n.d.^a^	n.d.^a^
*t*_1/2_ [s]	51	n.d.^a^	n.d.^a^	81	n.d.^a^	n.d.^a^
BG-OG (+)						
*k*_app_ [M^–1^s^–1^]	4.6 × 10^4^	2.3 × 10^4^	1.6 × 10^4^	3.0 × 10^4^	1.3 × 10^4^	0.5 × 10^4^
*t*_1/2_ [s]	75	148	212	116	275	636
SBG-OG (−)						
*k*_app_ [M^–1^s^–1^]	1.5 × 10^4^	n.d.^a^	n.d.^a^	0.9 × 10^4^	n.d.^a^	n.d.^a^
*t*_1/2_ [s]	231	n.d.^a^	n.d.^a^	398	n.d.^a^	n.d.^a^
SBG-OG (+)						
*k*_app_ [M^–1^s^–1^]	1.1 × 10^4^	0.6 × 10^4^	0.4 × 10^4^	0.8 × 10^4^	0.7 × 10^4^	0.4 × 10^4^
*t*_1/2_ [s]	321	533	821	420	521	904

a n.d. = not determinable via exponential fit from the measured data.

In the next step, we determined the self-labeling kinetics for SNAP-Y114ONBY­(-HT7) in their nonirradiated (−), photocaged state. Remarkably, binding of (S)­BG substrates remained in the early linear phase of the exponential association throughout the measurement, suggesting that the *k*_app_ values were too slow to be resolved in the chosen time frame of measurement (Figure S6). This confirmed our initial observation in the in-gel assays that the self-labeling of SNAP with (S)­BG substrates was efficiently hampered by ONBY in position Y114. Irradiation restored the ability of SNAP-Y114ONBY and SNAP-Y114ONBY-HT7 to bind BG-OG with *k*_app_ values of 2.3 × 10^4^ and 1.3 × 10^4^ M^–1^s^–1^, respectively (Table 1 and Figure S6). Likewise, binding of SBG-OG was recovered with *k*_app_ values of 0.6 × 10^4^ and 0.7 × 10^4^ M^–1^s^–1^, respectively. Notably, the self-labeling rates only achieved 51–60% for SNAP-Y114ONBY and 42–81% for SNAP-Y114ONBY-HT7 compared to SNAP and SNAP-HT7, respectively. Our previous studies indicated that this effect can be explained by the incomplete decaging of ONBY and particularly by the presence of undesired OABY, which is inert to irradiation. Indeed, native mass spectra indicated the presence of significant amounts of ONBY and OABY in the irradiated proteins (Figures S2 and S3). Moreover, decaging appeared to be more efficient in SNAP-Y114ONBY-HT7 than in SNAP-Y114ONBY due to the absence of the OABY.

Furthermore, we recorded the self-labeling kinetics for SNAP-Y158ONBY­(-HT7). Similar to SNAP-Y114ONBY­(-HT7), *k*_app_ values for the binding of (S)­BG-OG to the nonirradiated, photocaged proteins were too slow to be determined (Figure S6). However, irradiation restored the ability of SNAP-Y158ONBY and SNAP-Y158ONBY-HT7 to bind BG-OG with *k*_app_ values of 1.6 × 10^4^ and 0.5 × 10^4^ M^–1^s^–1^, respectively. Self-labeling rates with SBG-OG reached similar *k*_app_ values of 0.4 × 10^4^ M^–1^s^–1^ for both variants. As expected from SNAP-Y114ONBY­(-HT7), the self-labeling rates only achieved 36–39% for SNAP-Y158ONBY and 18–46% for SNAP-Y158ONBY­(-HT7) compared to SNAP and SNAP-HT7, respectively. These data again correlated with the decaging efficiencies determined via native MS (Figures S2 and S3), which confirmed that decaging was less efficient for SNAP-Y158ONBY­(-HT7) than for SNAP-Y114ONBY­(-HT7) due to initial decaging before irradiation and increased amounts of OABY. In line with our in-gel assays, our results therefore showed that both ONBY variants were effectively self-labeled after irradiation. In-gel assays and fluorescence polarization kinetics together demonstrated that SNAP-Y114ONBY­(-HT7) exhibited lower binding affinities toward (S)­BG substrates in its photocaged state, reached higher decaging efficiencies upon irradiation and as a result faster labeling rates in its decaged state compared to SNAP-Y158ONBY­(-HT7). For this reason, we focused on this variant, which we dubbed SNAPpa, in our further experiments.

### SNAPpa Facilitates Spatiotemporal Photocontrol of Cellular Targets

We next wondered how well SNAPpa performs in live-cell imaging, which is one of the main applications of SNAP-tagging. For this reason, we first transfected HEK293 cells transiently with a plasmid encoding for a SNAP construct with an N-terminally fused HT7 domain and a C-terminally fused nuclear localization signal (HT7-SNAP-NLS;^32^ Figure 3A) to set benchmark for labeling conditions and density. Nuclear staining was imaged on a confocal microscope in live cells using Hoechst for DNA staining, Halo-Tag-Ligand­(HTL)-SiR-d12 for HT7 staining, and BG-OG and BG-TMR for SNAP staining, as examples from our in vitro studies (Figure S7A). Owing to the unsatisfying amount of background noise, we, however, replaced the BG-OG and BG-TMR dyes for the recently designed neutral xanthene dye BG-SiR-d12 with highly improved fluorescence properties, as evident from bright signals in the nucleus (Figure 3B,C and Figure S7A). Accordingly, we swapped HTL-SiR-d12 for HTL-JF_549_. In parallel, we confirmed successful genetic code expansion in HEK293 cells by incorporating ONBY and as a control *trans*-cyclooct-2-en­(TCO)-lysine in position 39 of eGFP as a fluorescence readout with previously engineered aaRS/tRNA pairs that are orthogonal in eukaryotic expression systems (Figure S7B).^33^ This set the stage for expression of HT7-SNAPpa-NLS in HEK293 cells. When ONBY was neglected, we only observed HT7 labeling with HTL-JF_549_ in the cytosol as no NLS peptide was expressed (Figure 3B,D). Likewise, when ONBY was added in the dark, only HT7 could be imaged in the nucleus since ONBY incorporation facilitated NLS expression but prevented SNAPpa labeling (Figure 3B,E). However, preirradiation for 10 s using blue light (405 nm) and a high intensity of *I* = 10.1 W cm^–2^, which we anticipated is less toxic than irradiation with UV light, allowed for labeling of decaged SNAPpa (Figure 3B). In fact, following the far-red signal of BG-SiR-d12 (500 nM) in time course measurements for 30 min after blue-light application showed intensifying SNAPpa labeling signals from the nucleus (Figure 3F). Integrating the density of the BG-SiR-d12 signal followed by background correction using a different channel and normalization demonstrated an ∼3-fold signal increase without plateauing (Figure 3G), which represents the reaction kinetics between the decaged SNAPpa and the BG-SiR-d12 dye. For comparison, we also performed the time course experiment with BG-OG and BG-TMR but could not observe a significant increase in signal intensities over time, owing to the high background noise (Figure S7C). To test whether longer irradiation further improves decaging and hence labeling kinetics, we repeated the time course labeling experiments with BG-SiR-d12 after 60 s of irradiation. While we did not yield significantly higher signals after 30 min (Figure 3G, see the arrow), suggesting similar reaction kinetics, many cells were killed, which was quantified by the percentage of blebbing after half an hour (Figure 3H). Thus, we proceeded with only 10 s of irradiation for our following experiments. To further evaluate the decaging efficiency, we compared sets of HEK293 cells with either HT7-SNAPpa-NLS or HT7-SNAP-NLS and applied BG-SiR-d12 and HTL-JF_549_ for SNAP­(pa) and HT7 labeling, respectively (Figure 3I and Figure S7D). We then determined the intensity ratios of SNAP­(pa) and HT7 within the nucleus. Compared with the intensity ratios of SNAP, we found a larger signal spread for SNAPpa. Although some SNAPpa signals appeared below the signal spread of SNAP, which would indicate incomplete decaging, most were within the same range or higher, possibly due to bleaching of JF_549_ during irradiation (cf. Figure 3C–E), which was only performed for SNAPpa. This further indicates that ONBY has been largely converted to tyrosine. Moreover, using the same experimental approach, we were able to determine the efficiency of the ONBY incorporation in SNAPpa. For this, we compared the nuclear JF_549_ signal of HT7 fused to SNAPpa-NLS to the cytosolic signal of HT7 fused to a short fragment of SNAPpa as a result of premature termination of translation (Figure S7D). Remarkably, amber suppression was successful in 45–95% (means ± SD: 64 ± 11%) of cases (Figure 3J). Finally, we applied light of 405 nm to one position before recording a 9 × 9 image by stitching and observed SNAP-tagging only in the immediate vicinity of where decaging was triggered (Figure 3K). In contrast, all transfected cells were counterlabeled with JF_549_, demonstrating excellent spatiotemporal control of SNAP-tagging.

**Figure 3:**
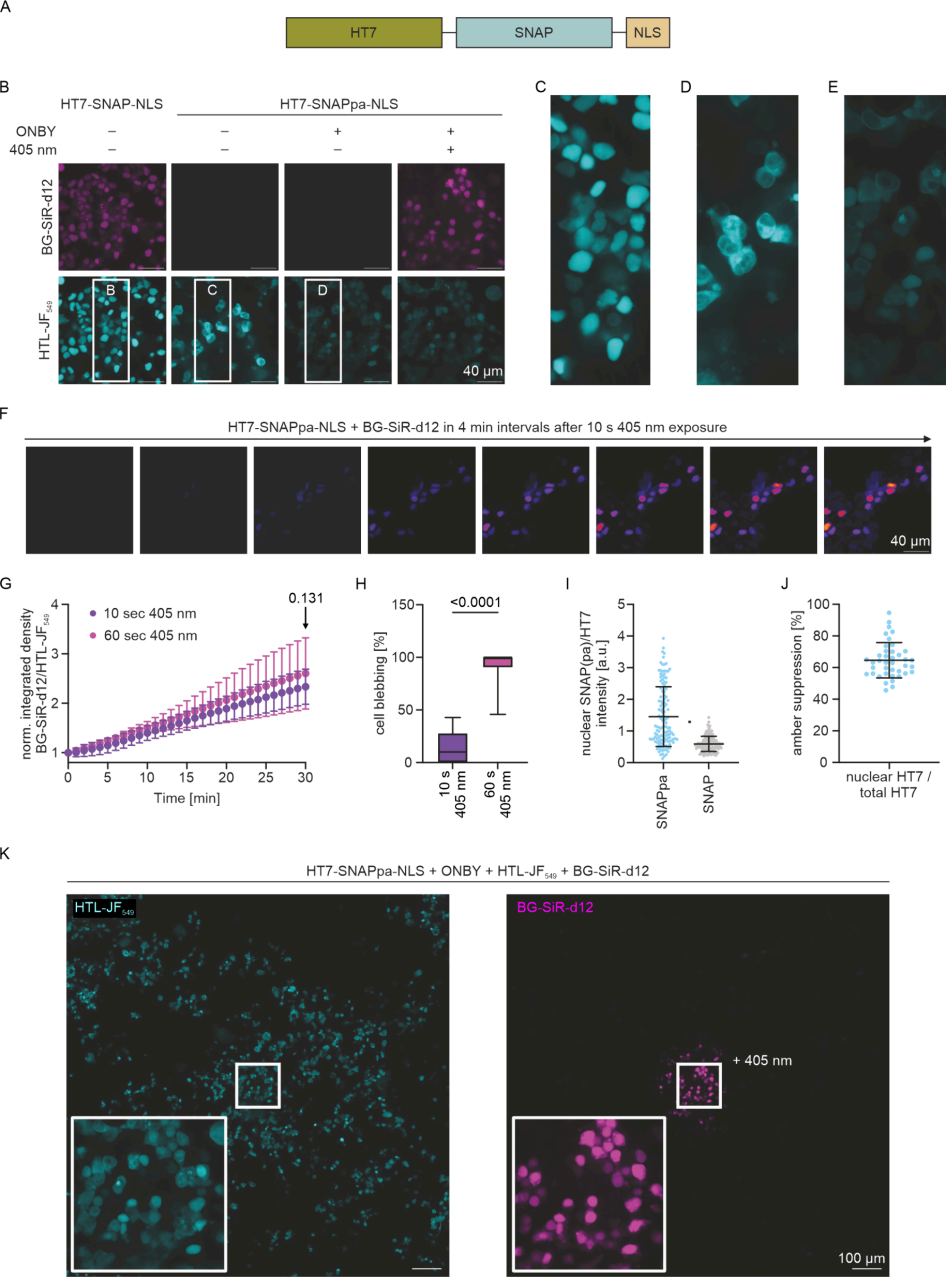
Photocaged SNAP-tag in live-cell imaging of cellular targets. (A) SNAP fusion construct with N-terminal HT7 and a C-terminal nuclear localization signal (NLS) for cell imaging studies in the cytosol and nucleus of HEK293 cells. (B) Confocal imaging of HT7-SNAP­(pa)-NLS transfected HEK293 cells, in which SNAPpa (localized in the nucleus) may only be labeled in the presence of ONBY and application of 405 nm light (10 s). HTL-JF_549_ served as a counterstain for HT7. (C–E) Zoom-ins from (B) showing localization of HT7 in the nucleus (C), in the cytosol (D), or both (E). (F) Time course labeling recordings of irradiated (405 nm, 10 s) SNAPpa in 4 min intervals. (G) Normalized (using the channel of JF_549_) integrated density of the BG-SiR-d12 signal shows similar fluorescence labeling after decaging with 10 or 60 s of irradiation. Statistics: means ± SD; *n* = 21; Student’s *t*-test for the last data point remains nonsignificant. (H) Counting blebbing cells 30 min after decaging with 405 nm shows significantly higher toxicity when using 60 s instead of 10 s of irradiation. Statistics: means ± SD; *n* = 21; Student’s *t*-test, unpaired. (I) Mean intensities of SNAP­(pa) stemming from the nucleus divided by mean intensity of nuclear HT7. Statistics: *n* = 129 and 136 nuclei for SNAPpa and SNAP, respectively. (J) Amber suppression efficiency assessed by dividing integrated HT7 density from the nucleus by total integrated density of whole cells. Statistics: *n* = 40. (K) Spatial control of HT7-SNAPpa-NLS decaging. HT7 labeling with HTL-JF_549_ served as an expression control.

### SNAPpa Allows for Labeling of Cell Surface Targets Employing No-Wash Protocols

We next aimed to address extracellular targets and used a SNAP-TM-HT7 fusion protein that localizes our SNAP on the extracellular side of a cell, while HT7 remains in the cytosol as they are separated by a single transmembrane (TM) domain (Figure 4A). For the first imaging, the cells were incubated with a cell-impermeable SBG-OG substrate for SNAP, with a cell-permeable HTL-SiR-d12 substrate for HT7 ^34^ as an expression control (Figure 4B, first column), and with Hoechst. For the second imaging, the cells were either kept in the dark or irradiated again with blue light (405 nm) in the presence of BG-Sulfo549. Application of cell-impermeable SBG-OG led to clear membrane staining, and saturation of SNAP was shown by chasing with cell-impermeable BG-Sulfo549, for which no signal was detectable. Next, we used SNAPpa-TM-HT7 for transfection and applied the same protocol. Without the addition of ONBY, no expression was detectable, as expected (Figure 4B, second column). However, when ONBY was added to the medium, we were able to stain HT7 but not SNAPpa (Figure 4B, third column), showcasing that the protein is expressed but not yet able to address the ONBY-containing tag. In the crucial experiment, we applied light of 405 nm in the presence of BG-Sulfo549, and after a washing step, we were able to clearly detect signals stemming from the cell surface after decaging (Figure 4B, fourth column). With this in hand, we next turned to image the SNAP-tagged glucagon-like peptide 1 receptor (GLP1R), a class B GPCR (G protein coupled receptor) that has found widespread attention as a target to combat obesity and type 2 diabetes mellitus.^35^ With our ongoing interest in labeling this receptor either by fluorophore-conjugated antagonistic peptides, the LUXendin family,^36,37^ or by endogenous CRISPR/Cas9-mediated endogenous SNAP-tag knock-in,^38^ we aimed to add this caged version to our toolbox. Indeed, using the same protocol as above, HEK293:SNAP-GLP1R was clearly labeled on the cell surface using SBG-OG and LUXendin651-d12 ^34^ (Figure 4C, first column). In contrast, when SNAPpa-GLP1R was used, the signal stemming from BG-Sulfo549 was only occurring when ONBY was present and light of 405 nm was applied, showcasing the generalizability of our method (Figure 4C, first to fourth column). With this potential spatiotemporal tool in hand, we used confocal spinning disk imaging on HEK293:SNAPpa-GLP1R cells in the presence of a low amount (25 nM) of BG-Sulfo549 that does not give rise to background signals and therefore neglects an additional washing step. In addition, we recorded images in 4 min intervals and integrated the labeling density of the images to deduce kinetics (Figure 4D,E). We considered this another advantage of caging SNAP, as time point zero is defined by applying light and is obtainable for fitting without technical challenges, e.g., adding substrates and setting up measurements. Indeed, we observed remarkable differences when comparing decaged SNAPpa-TM-HT7 versus SNAPpa-GLP1R with half-lives of 4.6 and 27.2 min, respectively (Figure 4F,G). Similar to the case for HT7-SNAPpa-NLS, we recorded a 9 × 9 image by stitching to demonstrate spatiotemporal control, and again, decaging was triggered (Figure 4H) only where light was applied with LUXendin651-d12 serving as a control for GLP1R’s orthosteric site.

**Figure 4:**
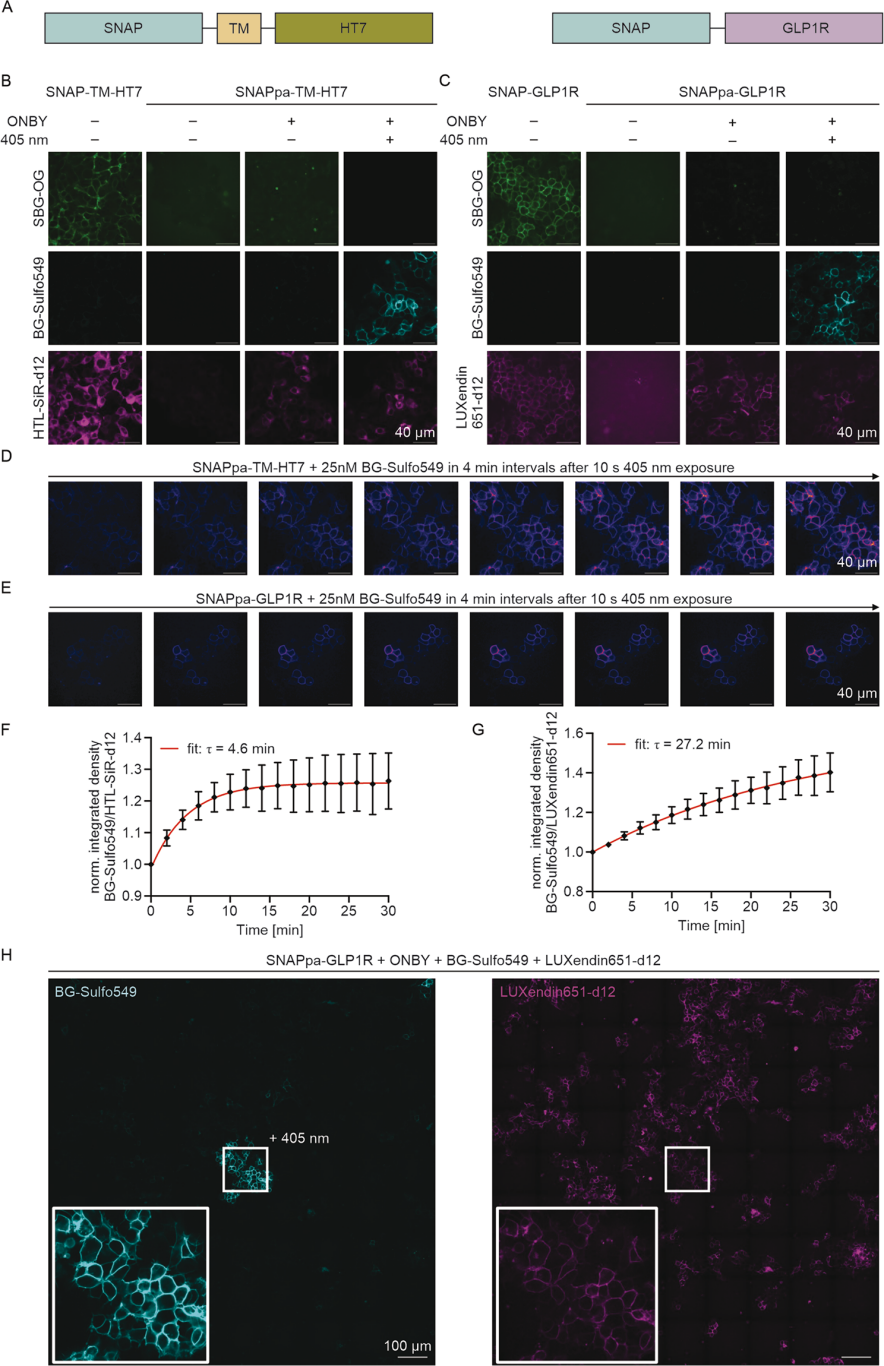
Photocaged SNAP-tag in live-cell imaging. (A) SNAP fusion construct with C-terminal HT7 separated by a transmembrane domain (TM), which localizes SNAP on the extracellular and HT7 at the cytosolic side of the cell membrane of HEK293 cells. (B) Confocal imaging of SNAP­(pa)-TM-HT7 transfected HEK293 cells, in which SNAPpa may only be labeled in the presence of ONBY and application of 405 nm light. HTL-SiR-d12 served as a counterbalance for HT7. (C) As for (A) but with SNAP­(pa)-GLP1R and LUXendin651-d12 as a counterstain. (D, E) Time lapse recordings of wash-free BG-Sulfo549 (25 nM) fluorescence after SNAPpa decaging in 4 min intervals. (F, G) Normalized integrated density from images in (C) and (D) to obtain labeling kinetics. Statistics: means ± SD; *n* = 3. (H) Spatiotemporal control of SNAPpa-GLP1R decaging. LUXendin651-d12 served as an expression control. Irradiation duration was 10 s for all decaging experiments.

## Discussion

In recent years, self-labeling protein tags have received increasing attention, particularly for live-cell imaging studies. Here, we further enhanced the attractiveness of this methodology with the option of spatiotemporally controlling the irreversible self-labeling reaction. Based on a human suicide enzyme, the SNAP-tag is one of the first, best studied, and most widely used examples of a self-labeling protein tag.^1–4^ Hitherto, the spatiotemporal control of the SNAP-tag has been realized by photoactivatable fluorescent substrates.^14–16^ However, photoactivation of the SNAP-tag reaction itself reduces synthesis efforts and simultaneously facilitates the use of a broad repertoire of substrates, which we established by the incorporation of a photocaged amino acid into SNAP. In this regard, the photocaged tyrosine ONBY offers well-established incorporation systems in prokaryotic and eukaryotic cells,^25,33^ is commercially available at reasonable prices, and has been applied in various research studies facilitating its usability.^39–43^ For these reasons, we considered the combination of SNAP and ONBY not only a good starting point for the light-induced activation of fluorescence labeling but also an easy to establish system for immediate implementation in cell imaging studies.

We have incorporated ONBY into all three tyrosine positions of SNAP, which has the advantage that the original tyrosine amino acids appear at these positions after light-induced decaging. While ONBY could be readily inserted into positions Y114 and Y158, we were unable to produce the respective SNAP-Y69ONBY variant. This confirms recent studies demonstrating that the efficiency of UAA incorporation is highly dependent on the position of incorporation.^44,45^ Irradiation of SNAP-Y114ONBY and SNAP-Y158ONBY variants, in fact, facilitated the photoactivation of the self-labeling reaction. Notably, in-gel assays showed that ONBY hampers the binding of BG substrates to SNAP more effectively in position Y114 than in position Y158. We correlate this observation to recent results showing that Y114 might be directly involved in substrate binding as well as the chemical step.^30,46^ For the most part, self-labeling of the SNAP-HT7 variants followed the same trend; however, signal intensities of the reactions with BG-TMR were comparable before and after irradiation. This indicates that photoactivation might potentially be limited for large SNAP fusion constructs in combination with BG substrates that possess very fast *k*_app_ values. Furthermore, fluorescence polarization kinetics revealed that after irradiation the self-labeling reaction was faster in the SNAP-Y114ONBY­(-HT7) variants than in the SNAP-Y158ONBY­(-HT7) variants, which could be explained by higher amounts of the aforementioned OABY in SNAP-Y158ONBY­(-HT7) as determined in native MS. In this context, we wondered whether the presence of OABY might be disadvantageous for the aspired application in cell studies. However, the emergence of OABY has so far only been detected after heterologous gene expression in prokaryotes and ascribed to the post-translational, enzymatic reduction in the cytosol as it can be minimized by transfer to the periplasm.^26^ This suggests that the OABY constitutes no major limitation for the spatiotemporal control of SNAP-tagging in mammalian cells, which is the primary application of our approach.

Overall, fluorescence polarization identified the most promising photoactivatable variant, SNAP-Y114ONBY, which we named SNAPpa. Similar to previous studies, the determined *k*_app_ values of SNAP for BG-OG and SBG-OG of 4.6 × 10^4^ and 1.1 × 10^4^ M^–1^s^–1^ were in good agreement with a previously determined range of 1.2 × 10^4^ to 4.3 × 10^5^ M^–1^s^–1^.^30^ The up to five-fold reduced *k*_app_ value of SBG-OG compared to BG-OG furthermore confirmed the recent observation that sulfonation at the C8 position of the BG leaving group slows down binding of the substrates.^13^ Although we were unable to obtain rate constants for the fully photocaged SNAPpa, comparison of the incompletely decaged SNAPpa with SNAP in initial presteady-state stopped-flow experiments provided first insights into how ONBY might affect the self-labeling reaction (extended Texts S1 and S2, Figure S8, and Table S1). Interestingly, all three steps included in *k*_app_, namely, binding, dissociation, and the chemical step, were hampered up to 17-fold for BG-OG. For SBG-TMR, only binding and dissociation rates were impaired up to seven-fold, implying that the mechanism of photoactivation might slightly differ between substrates.

We finally employed SNAPpa in live-cell imaging and demonstrated its feasibility for intracellular and extracellular POIs. Since prolonged UV light exposure or high intensities are cell toxic, we used blue light (405 nm) for decaging to reduce potential toxicities. Generally, phototoxicity is dependent on light intensity and duration of exposure, and accordingly, our results demonstrated increased blebbing of cells after 60 s of irradiation compared to 10 s of irradiation without a significant improvement of the SNAP signal. Toxicity may also stem from aromatic nitroso aldehyde breakdown products; however, ONBY has been extensively used in previous studies in cellular experiments to in vivo, and no adverse effects have been reported.^47^ These issues may be addressed by employing caged groups that can be liberated with longer wavelengths, leading to less reactive leaving groups.^48–50^ Of note, we observed that ONBY itself is toxic on cells and forms crystals at higher concentration that hamper imaging. For these reasons, we adjusted our protocol to maximize ONBY concentration in cellular media by adding a spatula tip of ONBY to DMEM/10% FBS, followed by sonication (10 min) and subsequent warming to 50 °C (10 min) before centrifuging the resulting suspension (13,000*g*; 10 min) and using the supernatant (1:1 dilution in full medium) for incubating cells. We found excellent viability of cells using this approach, as assessed by cell morphology (Figure S7B), and confirmed by cell viability assays WST-1 after overnight incubation with different dilutions (Figure S7E). With the possibility of no-wash labeling using cell-impermeable BG-Sulfo549 in low 25 nM concentrations, clean assessment of labeling kinetics after decaging was done. The fact that we observed substantial differences in labeling speed underpins the influence of the fusion protein, which needs careful validation in each case. With respect to other caged systems that liberate the BG moiety or photoinduce rearrangement reactions that generate a fluorophore with UV light, our approach uses blue light and is not limited by freely diffusing decaged compounds. Importantly, the gained spatiotemporal control offers many possibilities, apart from uncaging intracellular SNAP fusion, but also for stepwise uncaging enabling super-resolution imaging and compartment-selective uncaging for labeling with different cargos, for instance for delivering photoswitchable ligands that respond to distinct wavelengths.^51,52^ These are ongoing interests in our laboratories, alongside expanding this approach to other self-labeling tags, such as SNAPtag2,^53^ CLIP, and HaloTag variants,^54^ for fluorophore and pharmacophore^55^ targeting.

## Experimental Section

### Strains, Expression Vectors, Enzymes, and Chemicals

Expression cells *E. coli* BL21 Gold (DE3) were purchased from Agilent Technologies and further maintained following the manufacturer’s guidelines for the preparation of chemically competent cells. Expression plasmids pET51b_strep-SNAP-His10, pET51b_strep-SNAP-link-HT7-His10, SNAP-TM-HT7, SNAP-GLP1R, and mCherry-1xTag were previously designed and produced.^31,56,57^ The plasmids pEVOL_ONBY and *Mb*ONBYRS-pylT^33^ and MmPylRS­(AF)-tRNA-Pyl^M15^ (“GCEXpress”)^57^ for the incorporation of ONBY or TCO into proteins in *E. coli* or mammalian expression hosts have either been generated as described below or were provided by Alexander Deiters (University of Pittsburgh, Pittsburgh, PA, USA), respectively. *o*-Nitrobenzyl-*O*-tyrosine hydrochloride (96% EE) was purchased from Activate Scientific (AS22849), and TCO was purchased from SciChem (SC-8008). All other reagents and solvents were purchased in analytical grade or higher from commercial sources.

### Subcloning of pEVOL_ONBY

A pEVOL plasmid with two copies of a *Methanocaldococcus janaschii* TyrRS and its respective tRNA provided by Peter Schultz (Scripps Research Institute, La Jolla, CA)^23,58^ was used as a backbone. Both copies of the TyrRS were removed and replaced with *Bsa*I restriction sites to facilitate Golden Gate cloning.^59^ A previously designed *Mj*TyrRS gene for the incorporation of ONBY^25^ was codon-optimized for *E. coli*, synthesized with *Bsa*I restriction sites (GeneArt, Thermo Fisher Scientific), and cloned into the prepared pEVOL plasmid. The correct sequence of the entire plasmid was confirmed by Sanger sequencing (Microsynth Seqlab).

### Site-Directed Mutagenesis

Amber codons were introduced at the designated positions (Y69, Y114, and Y158) into pET51b_strep-SNAP-His_10_, pET51b_strep-SNAP-link-HT7-His_10_, SNAP-TM-HT7, and SNAP-GLP1R according to a modified protocol of the Q5 site-directed mutagenesis kit (New England Biolabs) with HPLC-purified primers (Metabion). Degradation of template DNA was performed by *Dpn*I digestion (New England Biolabs), and linear DNA was circularized in a combined phosphorylation-ligation reaction using T4 polynucleotide kinase and T4 DNA ligase (Thermo Fisher Scientific). Correct mutagenesis was confirmed by Sanger sequencing (Microsynth Seqlab) starting from the T7 promotor or terminator.

### Protein Expression and Purification

The SNAP and SNAP-HT7 proteins were produced by heterologous gene expression in *E. coli* BL21 Gold (DE3) cells (Agilent Technologies). The cells containing pET51b_strep-SNAP-His_10_ or pET51b_strep-SNAP-link-HT7-His_10_ were grown in 4 L of lysogeny broth (LB) medium at 37 °C until an OD_600_ of ∼0.6 was reached. Protein expression was induced with 0.5 mM isopropyl β-d-thiogalactopyranoside (IPTG) followed by incubation overnight at 16 °C. Cells were harvested by centrifugation and resuspended in 50 mM Tris-HCl (pH 7.3), 50 mM NaCl, and 10 mM imidazole. After lysis by sonication and repeated centrifugation steps, the supernatant was subjected to Ni^2+^-immobilized metal affinity chromatography (HisTrap FF Crude column, 5 mL, GE Healthcare). Proteins were eluted with a linear gradient of imidazole (10–750 mM), and fractions containing the protein of interest were identified by sodium dodecyl sulfate-polyacrylamide gel electrophoresis (SDS-PAGE), pooled, and concentrated. SNAP and SNAP-HT7 were further purified with a size-exclusion chromatography (SEC) column (Superdex 75 HiLoad26/260, GE Healthcare) by using 50 mM HEPES (pH 7.3) and 50 mM NaCl as the running buffer. Fractions were checked by SDS-PAGE for >80% purity, pooled, concentrated, and dripped into liquid nitrogen for storage at −70 °C.

For the production of SNAP-ONBY­(-HT7) variants, a slightly adjusted protocol was used. Thereby, the BL21 Gold (DE3) cells were cotransformed with the respective mutated version of pET51b_strep-SNAP-ONBY-His_10_ or pET51b_strep-SNAP-ONBY-link-HT7_His_10_ and pEVOL_ONBY. The cells were grown in 600 mL of terrific broth (TB) medium to an OD_600_ of 2–3 before gene expression was induced by adding 0.5 mM IPTG, 0.02% l-arabinose, and 0.4 mM ONBY. Cultures were incubated overnight at 30 °C, and proteins were purified in the dark as described above for the SNAP­(-HT7) protein.

### Native Mass Spectrometry

Protein samples were prepared in 200 mM ammonium acetate (AmOAc) at pH 7 (if not specified otherwise) and purified by size-exclusion chromatography on a Superdex Increase 200 10/300 column pre-equilibrated with 200 mM AmOAc at pH 7 (Cytiva, Massachusetts). All samples were sprayed at analyte concentrations ranging from 5 to 10 μM. Proteins were purified prior to the analysis and stored in the dark at −70 °C.

Approximately 4 μL of each solution was sprayed from borosilicate capillaries of ∼1 μm I.D. (B100-75-10, Sutter Instruments, California), prepared in-house using a micropipet puller (P-1000, Sutter Instruments), and fitted with a platinum wire. Mass spectra were acquired in positive mode on a Select series cyclic IMS (Waters, Wilmslow, UK). This device was fitted with a 32,000 *m*/*z* quadrupole filter and an electron-capture dissociation (ECD) cell in the transfer (post-IMS) region. The mass range was set to *m*/*z* 50–32,000 with a 1 s scanning rate. A 50:50 acetonitrile:water solution of a 20 μM cesium iodide (99.999%, analytical standard for HR-MS, Fluka, Buchs, Switzerland) was used as a calibration solution for the entire mass range. The quadrupole profile was set to an “automatic profile” to allow transmission of ions from a broad *m*/*z* range. The essential parameters of the mass spectrometer excluding IMS parts operating in “V-mode” were as follows: capillary voltage, 0.8–1.6 kV; sampling cone, 20 V; source offset, 30 V; source temperature, 28 °C; trap collision energy, 5 V; transfer collision energy, 5 V. Full lists of settings for the transmission of high-mass biomolecules are published elsewhere.^60^

### Circular Dichroism Analysis

Circular dichroism (CD) spectra in the far-UV range of 190–250 nm were recorded in a Jasco J-815 spectrophotometer with five accumulations. The spectra were measured with 7.5 μM SNAP variants or 2 μM SNAP-HT7 variants in 15 mM potassium phosphate (pH 7.5) in a 0.1 cm cuvette at 25 °C. The curves were smoothed using the Savitzky–Golay algorithm, and data were normalized to obtain the mean residue ellipticity as described previously.^61^

### Differential Scanning Calorimetry

SNAP variants (42 μM) or SNAP-HT7 variants (20 μM) were heated in degassed 50 mM HEPES (pH 7.3) and 50 mM NaCl from 25–130 °C at a ramp rate of 1 °C/min in a VPDSC differential scanning microcalorimeter (MicroCal, Malvern Instruments) with fixed reference and sample cells. The change in the heat capacity with an increase in the temperature was recorded. The proper equilibration of the calorimeter was ascertained by performing several buffer–buffer baselines. Overpressure was applied to prevent boiling above 100 °C while remaining in the liquid phase. DSC experiments were evaluated using Origin 2022 (OriginLab). The buffer signal was subtracted from the protein data, and baselines were corrected by using the spline interpolation option. Curves were then fitted with a nontwo-state model using the manufacturer’s software (MicroCal Analysis, embedded in Origin 7 SR4) to determine the denaturation midpoint temperature *T*_m_.

### In-Gel Fluorescence Analysis

BG substrates (7.5 μM) were mixed with SNAP variants (5 μM, if not specified otherwise) in 50 mM HEPES (pH 7.3), 50 mM NaCl, and 1 mM DTT. While one part of each sample was kept in the dark, the other part was irradiated (365 nm high-power LED ENGIN LZ4-44UV00-0000, 2 min). Both samples were incubated at 37 °C for 30 min in the dark before the reaction was quenched by the addition of 5× SDS sample buffer [1.25 M Tris-HCl (pH 6.8), 10% (v/v) glycerol, 5% (w/v) SDS, 5% (v/v) β-mercaptoethanol, and 0.01% (w/v) bromophenol blue] and heating to 95 °C for 3 min. The samples were then loaded onto a 13.5% SDS gel, and electrophoresis (in a chamber from Hoefer Pharmacia Biotech) was started by applying 300 V and 50 mA for 30 min in 25 mM Tris-HCl (pH 8.5), 0.1% (w/v) SDS, and 200 mM glycine. Subsequently, gels were scanned in a fluorescence gel scanner (Typhoon FLA 9500, GE Healthcare) with a FITC filter for (S)­BG-OG and a Cy3-filter for (S)­BG-TMR. After fluorescence analysis, protein bands were stained with Coomassie Brilliant Blue R250 and visualized in a GelDoc Go system (Bio-Rad). Image analysis was performed with ImageJ (W. Rasband, NIH). For double bands, only the upper band was included in the analysis.

### Fluorescence Polarization

Kinetic measurements were performed on a Jasco FP-6500 instrument by means of fluorescence polarization. BG substrates (50 nM) were prepared in 50 mM HEPES (pH 7.3), 50 mM NaCl, 1 mM DTT, and 100 ng/mL BSA in a submicrocuvette (16.100F/Q, Starna). The device-specific *G* value was determined for each reaction. The measurement was started before SNAP variants (200 nM in 50 mM HEPES (pH 7.3) and 50 mM NaCl) were added, and the fluorescence polarization signal was recorded over time [λ_ex_(OG) = 501 nm; λ_em_(OG) = 519 nm; λ_ex_(TMR) = 534 nm; λ_em_(TMR) = 570 nm; 25 °C; bandwidth, 10 nm; response time, 0.5 s; data pitch, 2 s; photomultiplier tube set between 350 and 370 V). Data of SNAP were exported to Origin 2022 and fitted with a monoexponential association model.

### Stopped-Flow Measurements

Labeling kinetics of SNAP and SNAP-Y114ONBY with the substrates BG-OG and SBG-TMR were measured by recording fluorescence emission changes over time using an SX20 stopped-flow instrument (Applied Photophysics) in a single-mixing configuration at 25 °C. The instrument was equipped with a Me-Xe-Arc lamp with monochromator wavelengths for excitation set at 485 nm paired with a cutoff filter at 495 nm for BG-OG and an excitation wavelength of 535 nm paired with a cutoff filter at 550 nm for SBG-TMR. Substrate BG-OG or SBG-TMR (25 nM) was mixed with an excess of SNAP protein (0.1–2.5 μM, concentrations refer to final concentrations in the observation cell) in a 1:1 volume ratio to observe the labeling reaction. To obtain the baseline, a 25 nM substrate was mixed with buffer only (50 mM HEPES, pH 7.3, 50 mM NaCl, and 1 mM DTT). Five to eight individual traces were recorded at each condition and averaged.

### Analysis of Stopped-Flow Data

Stopped-flow data were fitted to a two-step kinetic model (eqs 1 and 2)^30^ comprising reversible substrate binding (*k*_1_), unbinding (*k*_–1_) and irreversible covalent reaction (*k*_2_), with P representing the SNAP protein, S the substrate, PS the protein substrate complex, and PS* the protein–substrate conjugate.

$$P+S\overset{k_{1}}{\underset{k_{-1}}{⇌}}\mathrm{PS}$$ 1

$$\mathrm{PS}\overset{k_{2}}{\to }{\mathrm{PS}}^{*}$$ 2

The kinetic model was translated into differential eqs 3–6 using DynaFit software.^62^ In the DynaFit script for global fitting analysis (extended Text S1), the mixing delay of the stopped-flow instrument was set as a fixed parameter. The fluorescence of the free dye was set as offset for each time trace. It was assumed that the protein substrate complex and the reacted product are contributing equally to the fluorescence signal change. Hence, the response for both species was set equal in DynaFit and fitted as a differential response coefficient together with the kinetic constants *k*_1_, *k*_–1_, and *k*_2_. Standard deviations of fitted parameters were estimated with the Monte Carlo method.

$$\frac{d\left[P\right]}{dt}=-k_{1}\left[P\right]\left[S\right]+k_{-1}\left[\mathrm{PS}\right]$$ 3

$$\frac{d\left[S\right]}{dt}=-k_{1}\left[P\right]\left[S\right]+k_{-1}\left[\mathrm{PS}\right]$$ 4

$$\frac{d\left[\mathrm{PS}\right]}{dt}=k_{1}\left[P\right]\left[S\right]-k_{-1}\left[\mathrm{PS}\right]-k_{2}\left[\mathrm{PS}\right]$$ 5

$$\frac{d\left[{\mathrm{PS}}^{*}\right]}{dt}=k_{2}\left[\mathrm{PS}\right]$$ 6

The derived parameters *K*_d_ (dissociation constant) and *k*_app_ (apparent first-order reaction rate constant) were calculated by using the following equations:

$$K_{d}=\frac{k_{-1}}{k_{1}}$$ 7

$$k_{\mathrm{app}}=k_{1}\times \frac{k_{2}}{k_{2}+k_{-1}}$$ 8

### Cell Studies

HEK293T cells (70,000) were seeded on 8-well, PLL-coated ibidi dishes in full media (DMEM high-glucose, stable glutamax, 10% FCS) and (co)­transfected the next day with 400 ng of each plasmid (HT7-SNAP­(pa)-NLS, SNAP­(pa)-TM-HT7, and SNAP­(pa)-GLP1R ± *Mb*ONBYRS-pylT)^31,33^ using JETPrime (VWR) according to the manufacturer’s instructions. ONBY-containing medium was prepared by adding a spatula tip of ONBY into 1 mL of full media, sonicating for 10 min, warming to 50 °C for 10 min before spinning down 5 min at 13,000 rpm, and taking the supernatant and dilution 1:1 with full medial; the medium was exchanged 4 h post-transfection, and cells were incubated overnight at 37 °C. The next day, cells were stained with a combination of 500 nM BG-OG, 500 nM BG-TMR, 500 nM BG-SiR-d12, 500 nM HTL-JF_549_, 500 nM HTL-SiR-d12, 1 μM SBG-OG or 500 nM LUX651-d12, and 1 μM Hoechst33342 for 30 min at 37 °C as indicated in the figures, before washing once with media and imaging in FluoroBrite on a Nikon CSU-X1 using a 40× objective (air), equipped with an EMCCD camera (Andor AU-888), fast triggered acquisition, and a piezo Z-drive for fast imaging. An incubator (OKOLAB) was set to 37 °C with 5% CO_2_. For TCO incorporation, the cells were supplemented with the unnatural amino acid TCO*-A (25 mM) directly to the cell growth media. Therefore, the TCO*-A was diluted 1:4 with 1 M HEPES (pH 8.0) and added at a final concentration of 250 μM to the cells. Transfected cells were maintained in an incubator with 5% CO_2_ at 37 °C for 24 h and were subsequently labeled.

Lasers used were 488, 561, and 638 nm. Decaging was achieved by illumination with 405 nm (100% laser power for 10 s), and 1 μM BG-Sulfo549 was added for 10 min at 37 °C. One-time wash with FluoroBrite was performed, and imaging was continued in FluoroBrite. For spatiotemporal control of SNAPpa-GLP1R decaging, 25 nM BG-Sulfo549 was added to the medium, decaging was initiated, and imaging was performed with set ROIs in 2 min intervals. Image analysis was performed in a semiautomated manner. First, nuclei and cell boundaries were automatically segmented using the default nuclei and cyto3 models in Cellpose (v3.0.11). These segmentations were then manually refined by the user with the drawing tools available in the software. The final outlines were saved as a .zip archive of ROI files for use in Fiji. The size and mean fluorescence intensities of SNAPpa and HT7 for each segmented cell and nucleus were measured in Fiji using the standard measurement tools.

## Supplementary Material



## Notes

Additional figures associated with the results section such as raw data or processed data and the script for the global fitting analysis of SNAP stopped-flow labeling kinetics (PDF)
