# Supplementary material for: SNAPpa: A Photoactivatable SNAP-tag for the Spatiotemporal Control of Protein Labeling
Source: JACS Au. 2025 Jul 17;5(7):3589–602. doi: 10.1021/jacsau.5c00603 (PMC12308408; doi:10.1021/jacsau.5c00603)
Supplement: Supplementary file 1 [file au5c00603_si_001.pdf]

Supplementary Information

SNAPpa: A photoactivatable SNAP-tag for the spatiotemporal control of protein labeling

Sabrina Mandl,[1] Barbara Maiwald,[1] Elena Adlmanninger,[1] Ramona Birke,[2] Sandra Schlee,[1] Adam Pruška,[3] Philipp Bittner,[3] Renato Zenobi,[3] Tolga Soykan,[2] Gerti Beliu,[4,5] Johannes Broichhagen,[2*] Andrea Hupfeld[1*]

[1] Institute of Biophysics and Physical Biochemistry, Regensburg Center for Biochemistry, University of Regensburg, Universitätsstraße 31, D-93053 Regensburg (Germany)
[2] Leibniz-Forschungsinstitut für Molekulare Pharmakologie (FMP), Robert-Roessle-Str. 10, 13125 Berlin (Germany)
[3] Department of Chemistry and Applied Biosciences, ETH Zurich, CH-8093 Zurich (Switzerland)
[4] Regensburg Center for Ultrafast Nanoscopy (RUN), Bioimaging, Faculty of Chemistry and Pharmacy, University of Regensburg, 93040 Regensburg (Germany)
[5] Rudolf Virchow Center, Research Center for Integrative and Translational Bioimaging, University of Würzburg, 97080 Würzburg (Germany)
* andrea.hupfeld@ur.de, phone: +49 941 943 7439
broichhagen@fmp-berlin.de, phone: +49 30 94793 171

Table of Contents

Figure S1. 2
Figure S2. 3
Figure S3. 4
Figure S4. 5
Figure S5. 6
Figure S6. 7
Figure S7. 8
Extended Text S1. 9
Table S1. 10
Figure S8. 11
Extended Text S2. 12

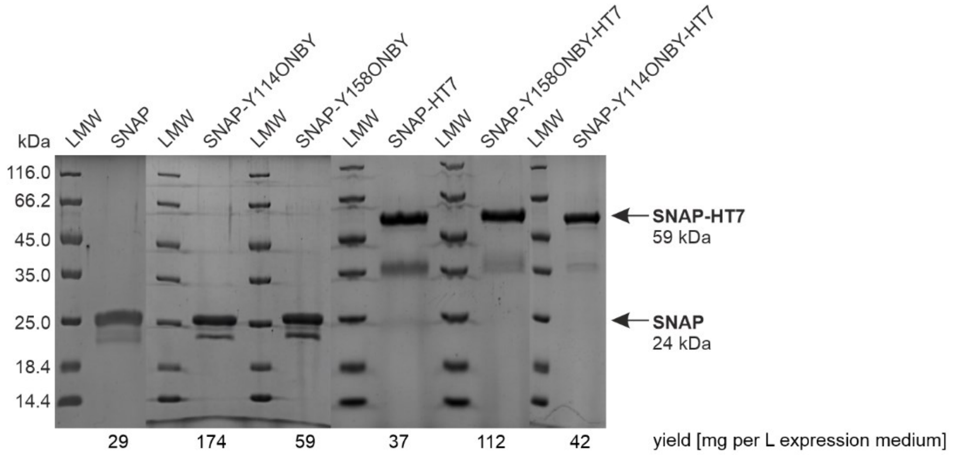

Figure S1. SDS-PAGE of 3 μg protein confirming >90% purity for SNAP variants and >80% purity for SNAP-HT7 variants. Additional bands at ~23 kDa for all SNAP variants and ~36 kDa for all SNAP-HT7 variants resulted from proteolytic cleavage as previously observed.[1] Yields in mg per liter expression medium are provided below the picture. LMW: low molecular weight marker.

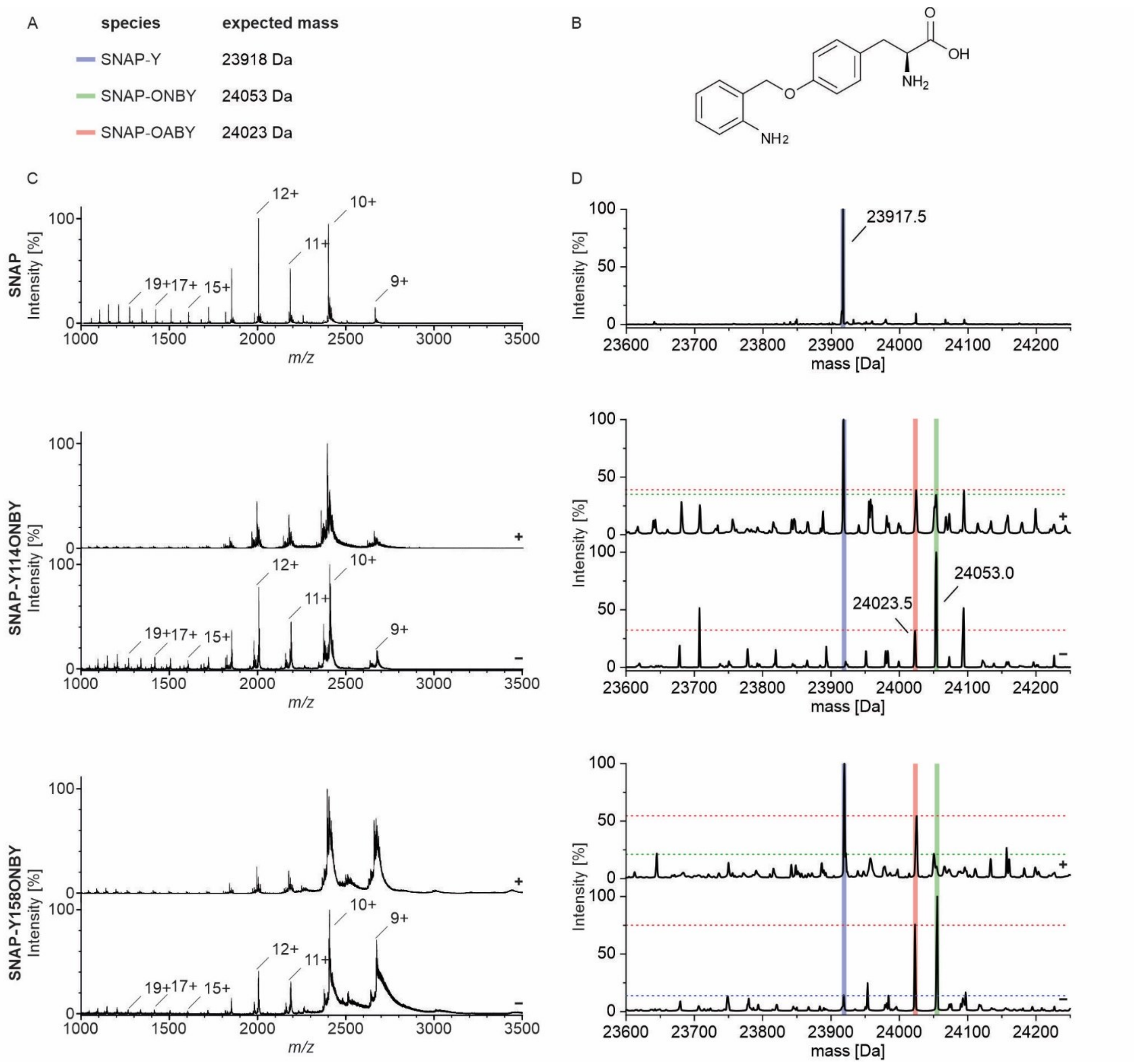

Figure S2. Native MS analysis of SNAP variants. A) Expected masses (excluding methionine at position 1 and including a Zn^{2+} ion) of the SNAP species containing tyrosine (Y), ONBY or OABY at position 114 and/or 158. B) Structure of o-aminobenzyl-*O*-tyrosine (OABY). C) Native MS spectra of SNAP, and both ONBY variants in their non-irradiated, photocaged ("−") and irradiated, decaged ("+") state (365 nm, 2 min). D) Deconvoluted native MS spectra highlighting species that contain tyrosine (blue), ONBY (green) or OABY (red). Dashed lines simplify the direct comparison of peak intensities of each species.

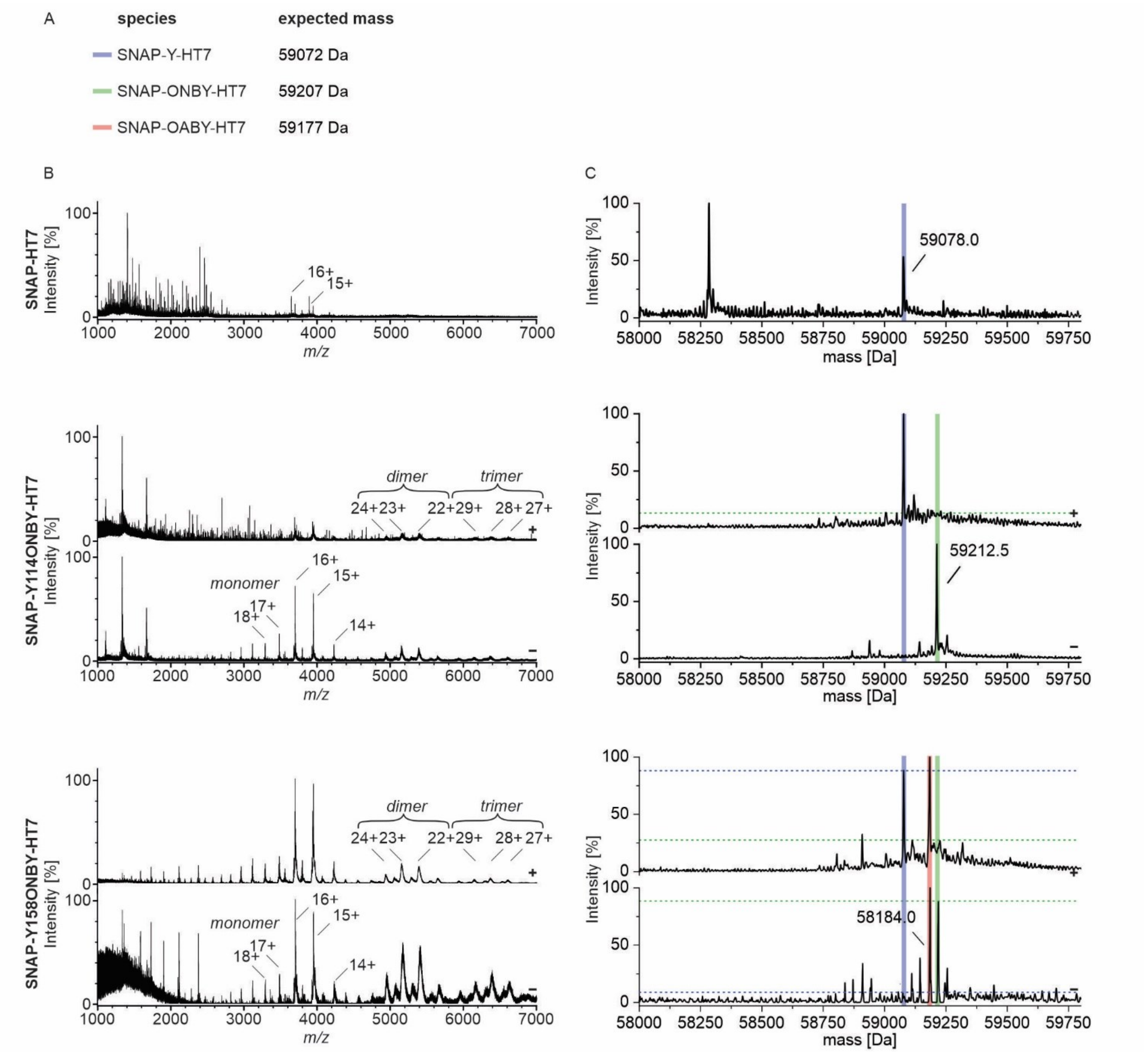

Figure S3. Native MS analysis of SNAP-HT7 variants. A) Expected masses (excluding methionine at position 1 and excluding a Zn^{2+} ion) of the SNAP-HT7 species containing tyrosine (Y), ONBY or OABY at position 114 and/or 158. B) Native MS spectra of SNAP-HT7, and both ONBY variants in their non-irradiated, photocaged ("−") and irradiated, decaged ("+") state (365 nm, 2 min). Note that SNAP-HT7 comprises an unidentified species missing 796 Da, which is likely the result of proteolytic cleavage. C) Deconvoluted native MS spectra highlighting species that contain tyrosine (blue), ONBY (green) or OABY (red). Dashed lines simplify the direct comparison of peak intensities of each species.

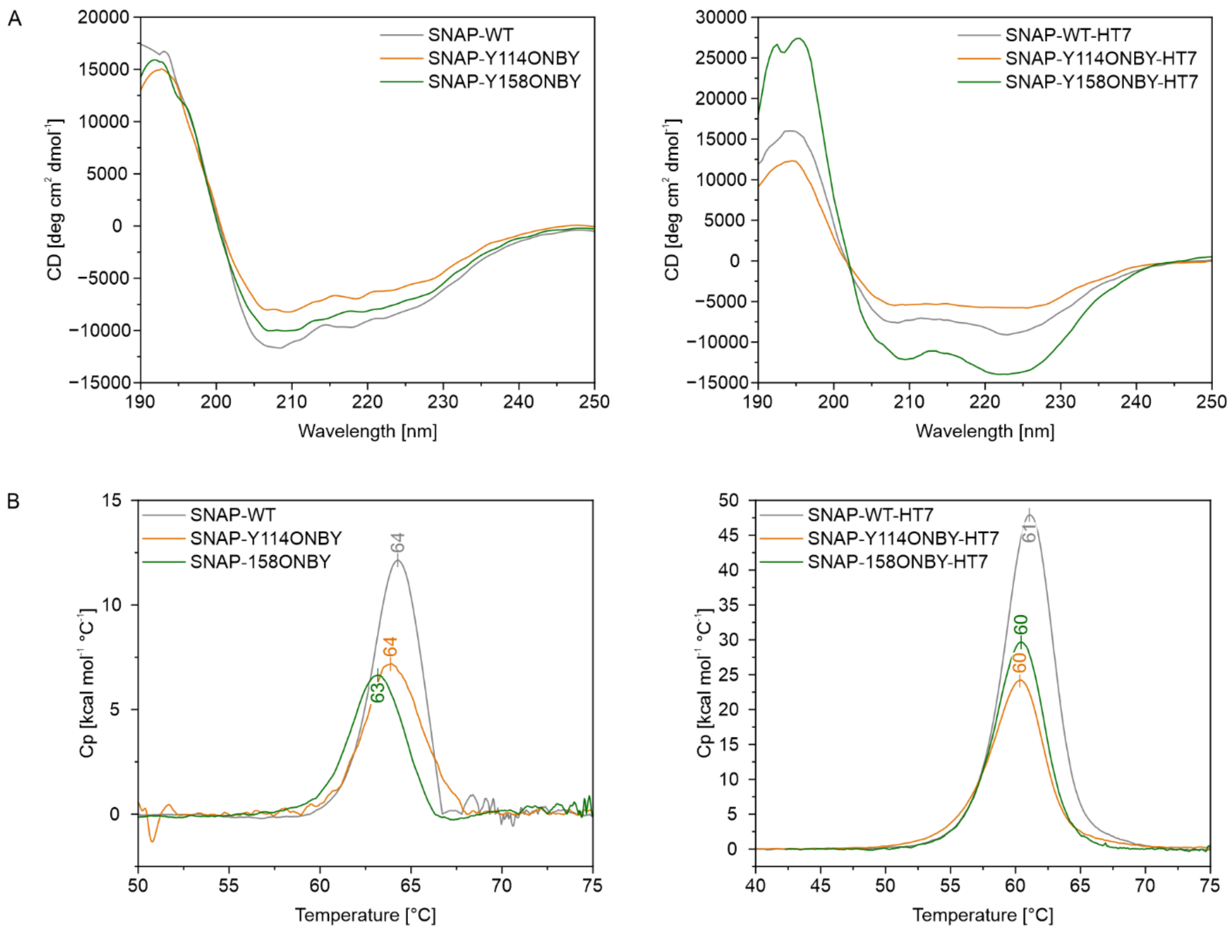

Figure S4. Structural integrity and thermal stability of the SNAP and SNAP-HT7 variants. A) Far-UV circular dichroism spectra indicate an intact overall fold of each SNAP or SNAP-HT7 variant. B) Differential scanning calorimetry (DSC) measurements indicate that the thermal stability is retained after incorporation of ONBY with denaturation midpoints T_m of 63–64°C for the SNAP variants and 60–61°C for the SNAP-HT7 variants. Note that different signal intensities result from the determination of protein concentrations via the Bradford assay, which is less accurate than the spectroscopic determination at 280 nm but is required because of the absorbance signal of ONBY at 280 nm.

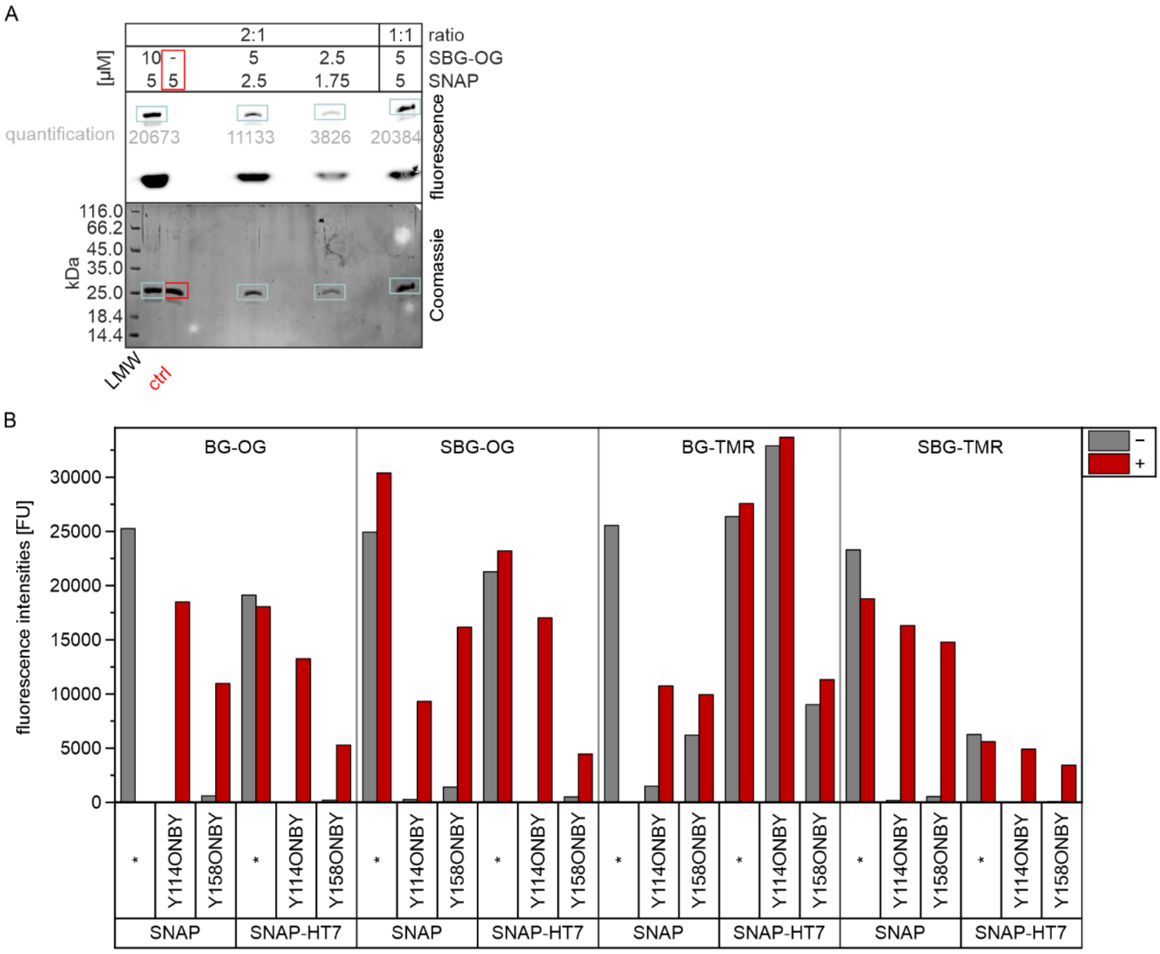

Figure S5. Self-labeling activity of SNAP(-HT7) ONBY variants using in-gel fluorescence analysis. A) SDS-PAGE of SNAP applied in various concentrations in the absence (“ctrl”; red) or presence (blue) of SBG-OG verifies that the measured fluorescence intensities of the retained signals reflect the amounts of self-labeled SNAP protein. Since we detected only a minimal difference in the fluorescence signal using a 1:1 ratio of SNAP and SBG-OG compared to a 1:2 ratio, we decided to apply a 1:1.5 ratio in all subsequent in-gel assays. B) Bar graph summarizing the fluorescence intensities of non-irradiated, photocaged (“−“) and irradiated, decaged (“+”) (365 nm, 2 min) variants determined in the in-gel assays (**Figure 2B**).

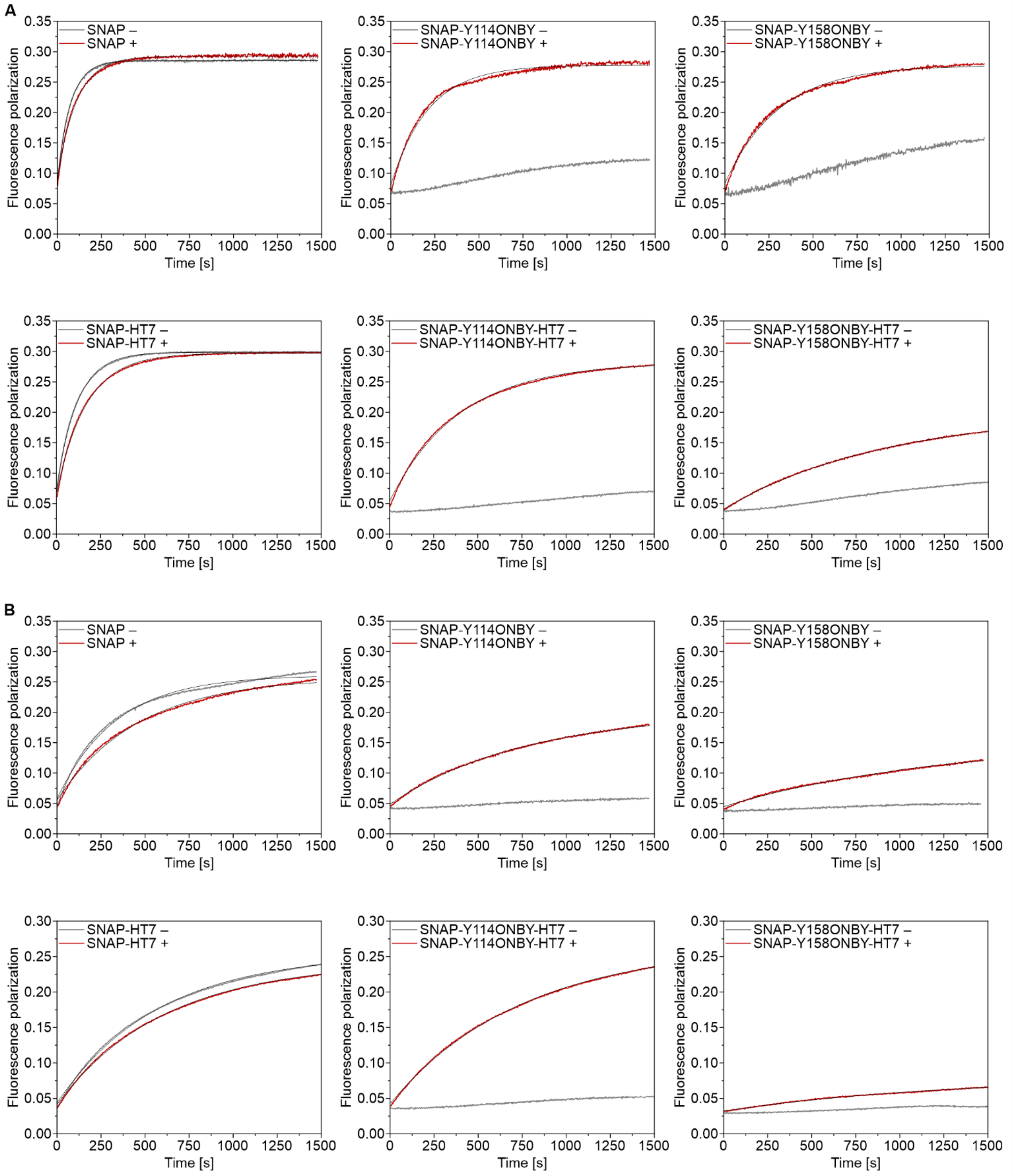

Figure S6. Fluorescence polarization measurements of SNAP(-HT7) variants with BG-OG (A) or SBG-OG (B). The signals confirm that non-irradiated, photocaged (−; grey) proteins exhibit a reduced self-labeling efficiency. Irradiation with 365 nm for 2 min to generate the decaged proteins (+; red) restores the self-labeling activity. The raw data were fitted with a mono-exponential fit to obtain k_{app} values (black).

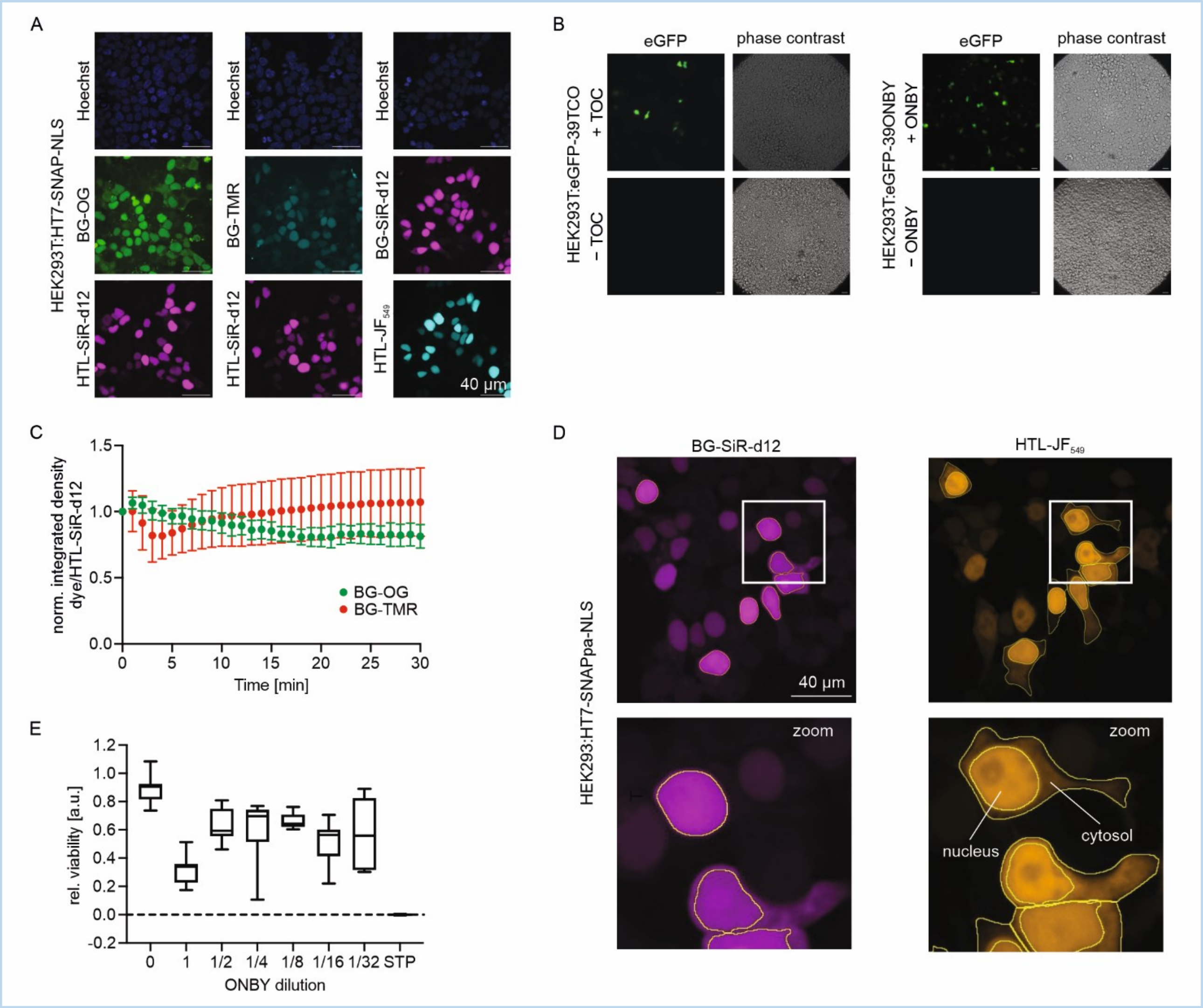

Figure S7. Dye testing, validation of unnatural amino acid incorporation and cell viability. A) Images of HEK293 cells transfected with HT7-SNAP-NLS to screen for dyes, where BG-SiR-d12 outperforms BG-OG and BG-TMR. B) HEK293 cells co-transfected with eGFP-39TAG and the respective synthetase while incubated with TCO or ONBY yields green fluorescing cells, accounting for successful amber suppression. C) Time course experiment of HEK293 cells transfected with HT7-SNAPpa-NLS, 10 s uncaging with 405 nm for labelling with either BG-OG or BG-TMR and integrating density to be divided by integrated density of HT7 signals stemming from SiR-d12. D) Representative image showing mask application for nuclear import. Masks were set by applying Hoechst33342 after the experiments. E) Cell viability WST-1 assay with different dilution of ONBY. 1/2 dilution has been used in cell experiments for uncaging. Staurosporine (STP) served as positive control. 2 biological replicates in quadruplicates. Min to max box and whisker.

Extended Text S1. Initial pre-steady state kinetic evaluations of SNAPpa self-labeling

We focused on SNAP without HT7 and tested in stopped-flow measurements whether the binding of different fluorophore substrates is accompanied by a change in fluorescence emission. We performed initial experiments by following the change of fluorescence over time that was induced by the mixing of the BG substrates with a large excess of SNAP. For the substrates BG-OG, SBG-OG and SBG-TMR a change in fluorescence was observed upon binding to SNAP, whereas no change in fluorescence emission occurred for the substrate BG-TMR. Interestingly, binding of (S)BG-OG was accompanied by a decrease, while binding of SBG-TMR was associated with an increase in fluorescence emission. For the two fastest binding substrates BG-OG and SBG-TMR stopped-flow time traces were recorded at different concentrations of SNAP(pa) under pseudo-first order reaction conditions (**Figure S8**). All time curves showed simple exponential progressions and data sets were fitted in a global analysis to a two-step kinetic model that allowed the determination of the three kinetic parameters k_1, k_{-1} and k_2 independently. The reaction steps in the kinetic model comprise reversible substrate binding (k_1), unbinding (k_{-1}), and the irreversible covalent reaction (k_2). Accordingly, the K_d value ($K_d = k_{-1}/k_1$) and the apparent rate constant k_{app} [$k_{app} = k_1 * k_2/(k_2+k_{-1})$] for the self-labeling reaction were calculated from the individual rate constants obtained in the global fitting procedure (**Table S1**).

With regard to the SNAP self-labeling kinetics, we observed that k_1 values were rather similar for BG-OG (k_1 = 5.8×10^4 M^{-1}s^{-1}) and SBG-TMR (k_1 = 7.9×10^4 M^{-1} s^{-1}), while the unbinding reaction of BG-OG (k_{-1} = 0.0018 s^{-1}) was about 60-fold slower compared to SBG-TMR (k_{-1} = 0.108 s^{-1}). This results in an approximately 44-fold higher K_d-value, i.e. a significantly weaker binding affinity for the substrate SBG-TMR. However, since the rate of the irreversible covalent reaction step was 5-fold faster for SBG-TMR (k_2 = 0.138 s^{-1}) than for BG-OG (k_2 = 0.029 s^{-1}), the k_{app}-values for the overall self-labeling reactions were again very similar to each other [k_{app}(BG-OG) = 5.5×10^4 M^{-1}s^{-1}, k_{app}(SBG-TMR) = 4.4×10^4 M^{-1}s^{-1}].

In stopped-flow measurements with SNAPpa in its non-irradiated, photocaged state, fluorescence changes were too small for quantitative analysis. In contrast, the reaction of BG-OG and SBG-TMR with irradiated, decaged SNAPpa generated analyzable signal amplitudes in stopped-flow fluorescence measurements, that differed from the reaction process of the unmodified SNAP. Since we have to assume that SNAPpa has not been completely decaged, this analysis allows conclusions to be drawn as to which reaction steps and associated rate constants are affected by residual ONBY.

For the substrate BG-OG, a comparison of the rate constants for the reaction with SNAP and irradiated SNAPpa showed an approximately 5-fold reduction in k_1, accompanied by a 2-fold increase in k_{-1}, which resulted in a 10-fold increase in the K_d value [K_d(SNAP) = 0.031 µM, K_d(SNAPpa) = 0.30 µM]. Notably, we also observed an approximately 14-fold reduced value of k_2 for irradiated SNAPpa (k_2 = 0.0017 s^{-1}), indicating that both the binding affinity and the chemical reaction step were affected by ONBY in the labeling reaction with BG-OG.

For the substrate SBG-TMR, an approximately 2-fold reduced k_1 and 4-fold increased k_{-1} resulted in an approximately 7-fold deterioration in affinity [K_d(SNAP) = 1.4 µM, K_d(SNAPpa) = 8.9 µM]. k_2 was only marginally affected, indicating that ONBY, in the case of SBG-TMR, only impairs substrate binding but not the covalent reaction step.

Table S1. Self-labeling kinetics of SNAP and irradiated, decaged ("+") SNAPpa (365 nm, 2 min) with BG-OG and SBG-TMR as determined via stopped flow kinetics.

	SNAP	SNAPpa
**BG-OG (+)**		
k_1 [M^{-1} s^{-1}]	$(5.84 \pm 0.01)\times10^{4}$	$(1.23 \pm 0.00)\times10^{4}$
k_{-1} [s^{-1}]	$(1.82 \pm 0.14)\times10^{-3}$	$(3.68 \pm 0.15)\times10^{-3}$
k_2 [s^{-1}]	$(2.90 \pm 0.22)\times10^{-2}$	$(1.69 \pm 0.65)\times10^{-3}$
k_{app} [$M^{-1}s^{-1}$][a]	5.50×10^{4}	3.87×10^{3}
K_d [µM][c]	0.031	0.300
**SBG-TMR (+)**		
k_1 [M^{-1} s^{-1}]	$(7.92 \pm 0.35)\times10^{4}$	$(4.99 \pm 0.38)\times10^{4}$
k_{-1} [s^{-1}]	$(1.08 \pm 0.16)\times10^{-1}$	$(4.46 \pm 0.59)\times10^{-1}$
k_2 [s^{-1}]	$(1.38 \pm 0.11)\times10^{-1}$	$(2.00 \pm 0.10)\times10^{-1}$
k_{app} [$M^{-1}s^{-1}$][a]	4.44×10^{4}	1.54×10^{4}
K_d [µM][c]	1.36	8.94

[a]Defined as $k_{app} = k_1 \times \frac{k_2}{k_2+k_{-1}}$. [c]Defined as $K_d = \frac{k_{-1}}{k_1}$.

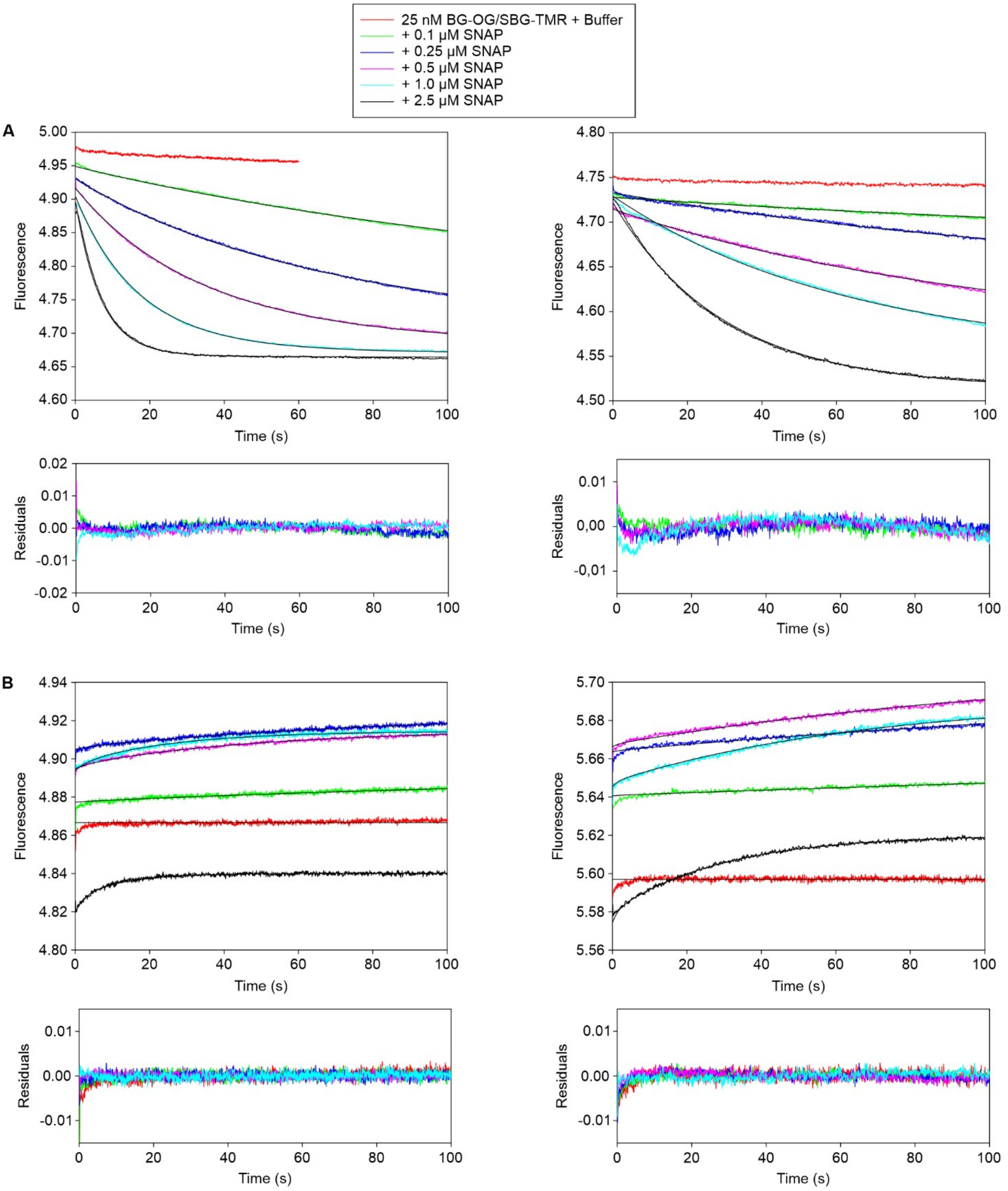

Figure S8. Characterization of SNAP pre-steady state labeling kinetics. Fluorescence emission traces (colored lines) and fitted curves (thin black lines) of SNAP labeling with BG-OG (A) and SBG-TMR (B) are shown. Kinetics were recorded with a stopped-flow device by following the fluorescence emission over time after mixing the indicated (365 nm, 2 min) concentrations of SNAP and the respective ligand in a 1:1 stoichiometry. Emission traces obtained with unmodified SNAP (left subpanels) and SNAP-Y114ONBY (right subpanels) are compared. The residuals reflect the difference between measured data and fit curves. Data were fitted in a global fitting procedure to a two-step kinetic model using the program DynaFit.

Extended Text S2. Script for the global fitting analysis of SNAP stopped flow labeling kinetics (DynaFit)

```
[task]
   data    = progress
   task    = fit
   model   = two-step
[mechanism]
   P + S  <===> PS     :   k1    k-1
   PS       ---> PS*     :   k2
[constants] ;units: µM, sec
   k1 = 0.06 ?
   k-1 = 0.005 ?
   k2 = 1 ??
[concentrations] ; µM
   S = 0.025
[responses] ; differential response coefficient
  PS* = -12 ?
  PS = 1 * Z
[data]
   directory   ./path to data
   extension   csv
   delay     0.01
   file BG-OG_buffer | conc P = 0 | offset auto ?
   file BG-OG_01      | conc P = 0.1 | offset auto ?
   file BG-OG_02      | conc P = 0.25 | offset auto ?
   file BG-OG_03      | conc P = 0.5 | offset auto ?
   file BG-OG_04      | conc P = 1.0 | offset auto ?
   file BG-OG_05      | conc P = 2.5 | offset auto ?
[output]
   directory  ./path to output folder
[end]
```

References

(1) Birke, R.; Ast, J.; Roosen, D. A.; Lee, J.; Roßmann, K.; Huhn, C.; Mathes, B.; Lisurek, M.; Bushiri, D.; Sun, H.; Jones, B.; Lehmann, M.; Levitz, J.; Haucke, V.; Hodson, D. J.; Broichhagen, J. Sulfonated red and far-red rhodamines to visualize SNAP- and Halo-tagged cell surface proteins. *Org. Biomol. Chem.* **2022**, *20* (30), 5967–5980. DOI: 10.1039/d1ob02216d.
